# European Rabbit Invasion in a Semi-Arid Ecosystem of Chile: How Relevant Is Its Role in Food Webs?

**DOI:** 10.3390/life13040916

**Published:** 2023-03-31

**Authors:** Patricia Gübelin, Jennifer Paola Correa-Cuadros, María Isidora Ávila-Thieme, Gabriela Flores-Benner, Melanie Duclos, Mauricio Lima, Fabián M. Jaksic

**Affiliations:** 1 Departamento Ecología, Facultad de Ciencias Biológicas, Pontificia Universidad Católica de Chile, Santiago 7820436, Chile; patricia.prez@uc.cl (P.G.); msavila@uc.cl (M.I.Á.-T.); gvflores@bio.puc.cl (G.F.-B.); mlima@bio.puc.cl (M.L.); fjaksic@bio.puc.cl (F.M.J.); 2 Center of Applied Ecology and Sustainability (CAPES), Santiago 8331150, Chile; mduclos@bio.puc.cl; 3 Instituto Milenio en Socio-Ecología Costera (SECOS), Santiago 8331150, Chile; 4 Advanced Conservation Strategies (ACS), Midway, UT 84049, USA; 5 CIS-UNAB Centro de Investigación para la Sustentabilidad, Santiago 8370255, Chile

**Keywords:** community feedbacks, conservation targets, invasive species, management practices, network models, *Oryctolagus cuniculus*, predator-prey relationships, trophic interactions

## Abstract

The European rabbit (*Oryctolagus cuniculus*) is one of the main invasive species in Chile, where it became naturalized ca. 150 years ago. Their high reproductive capacity, lack of specialist predators, and great adaptability favored the settlement of rabbits in diverse mainland and island ecosystems of the country. Recently, rabbits have become central players in semi-arid ecosystems, such as those represented in Las Chinchillas National Reserve in north-central Chile. We undertook to analyze the place and role of rabbits in the food web of that Reserve, based on a bibliographic review and long-term annual data gathered from 1987 until 2022 (36 years). Results showed that the network comprised 77 species, where 69% were primary producers (plants), 18% were mid-level consumers (herbivores), and 13% were top-level consumers (predators). The most connected species in the food web was the rabbit, which positively or negatively affected the species interacting with it. Predators such as *Galictis cuja*, *Geranoaetus polyosoma*, *Leopardus colocolo*, and *Puma concolor*, and the scavenger *Vultur gryphus*, could be negatively affected by an eventual decrease (natural or human-caused) in the rabbit population of the Reserve. To the contrary, primary producers such as *Oxalis perdicaria*, *Plantago hispidula*, *Schizanthus parvulus*, *Senna cumminggi*, and *Tropaeolum azureum* could be positively affected by an increase in their biomass in response to a decrease in rabbits, favoring native rodents. We consider that analyzing the rabbit-centered food web and its impacts on native interacting species allows a better understanding of the relevance of invasive species in the local community, providing conceptual tools for rabbit management.

## 1. Introduction

Human introduction of species has facilitated their expansion to regions beyond the limits of their dispersal capacities [1]. Invasive species are one of the main causes of biodiversity loss, affecting natural ecosystems, productive systems, and human health; reducing their impacts is, therefore, one of conservation management’s main goals [2]. The spread of such species into new areas involves three phases: (i) settlement, which occurs after its arrival and ends with local extinction or naturalization, if the population generates viable progeny; (ii) expansion, where the settled population increases its abundance and occupies all favorable habitats; and (iii) persistence, where the species occupies all accessible habitats [3], integrating with local communities and generating changes in ecosystem processes [4].

The introduction of alien species to Chile probably started in the pre-Columbian period, contributing to the expansion of numerous plants, such as the shrub *Acacia caven* [5]. Later, under the Spanish colonization of the 16th century, commerce developed further, allowing the introduction of new species into the Chilean ecosystems, such as the European rabbit (*Oryctolagus cuniculus*) [6]. This rabbit is on the list of the world’s 100 worst invasive alien species [7] and is among the seven most harmful invasive species present in Chile [8]. It was introduced as a caged animal in the 18th century for commercial purposes (pelt, fur, and meat) [6]. Captive rabbits were released to the wild, and others escaped, causing subsequent outbreaks in central Chile, and expanded geographically southward to Los Lagos Region and northward to Atacama Region [9,10]. A separate escape/release occurred in the Magallanes Region [6,11,12,13].

As a consequence of the preceding, rabbits damage agricultural, forestry, and livestock operations, and also interfere with Chilean ecosystems’ structure, composition, and function [8]. For instance, rabbits modify the spatial distribution and decrease the survival rate of native plant species, facilitate the dispersal of invasive plants thus driving native plants to local extinction, generate soil erosion, and prevent the normal renewal and successional processes of plant communities [6,13,14,15,16,17,18,19]. Rabbits in Chile use the open spaces within shrublands, modifying the landscape to their needs [15,20,21,22]. They prefer to eat native perennial herbs, removing unsheltered herbs and shrub seedlings, thus forestalling their reproduction [14,16,23,24]. The rapid spread and broad distribution of rabbits is attributed to their high population growth rate and reproductive capacity, with a gestation of only 30 days and the females’ ability to go into an estrous cycle while nursing their young [25]. Under wild conditions in Australia and Chile, females can have several litters a year with up to seven offspring each [8,26], and this renders the rabbit a successful invasive species.

According to Jaksic et al. (1979, 1981), Jaksic and Soriguer (1981), Jaksic and Ostfeld (1983), Jaksic and Yáñez (1983), and Jaksic (1986), rabbits in Chile are mostly preyed upon by native carnivores such as foxes (*Lycalopex culpaeus* and *L. griseus*), cats (*Puma concolor*, *Leopardus guigna*, and *L. colocolo*), skunks (*Conepatus chinga*), and grisons (*Galictis cuja*); and by alien carnivores such as mink (*Neovison vison*) and domestic cats and dogs [11,20,27,28,29,30]. Additionally, they are hunted by kestrels, eagles, and hawks (*Falco sparverius*, *Geranoaetus melanoleucus*, *G. polyosoma*, and *Parabuteo unicinctus*); and by owls (*Athene cunicularia*, *Bubo magellanicus*, and *Tyto alba*). Still, this wealth of predators does not seem to be able to keep rabbit populations at low abundance for any prolonged time [11,21,22].

Usually, specialist predators can regulate prey abundance preventing population increases and inducing population cycles [31,32,33], but rabbits in Chile do not have specialized predators [8,34,35]. Often, generalist predators cannot control rabbits at high densities because their consumption rate curve rapidly saturates, and the population growth rate of rabbits is greater than the predators’ growth rate [8,11,26,36,37]. Additionally, generalist predators easily switch between rabbits and abundant alternative prey, basically rodents, contributing to relaxing the already weak predator control [29,30,31,38]. Indeed, several authors point out that generalist predators can keep mammalian prey at low densities only when the latter have already been affected by droughts, heavy snowfalls, or diseases [34,35,39,40,41,42,43,44].

The first studies on rabbit predation in Chile (conducted in the 1970s) showed that this species was a minority fraction in the diets of *Athene cunicularia*, *Bubo magellanicus*, *Geranoaetus melanoleucus*, *G. polyosoma*, *Lycalopex culpaeus*, *L. griseus*, *Parabuteo unicinctus*, and *Tyto alba* [45,46,47,48,49,50]. Such low predation upon rabbits was attributed to native predators not having yet adapted to hunt for this recently introduced prey [21,22,37,51]. Indeed, Jaksic (1986) hypothesized that those native predators were accustomed to the simple escape behavior of their native prey (which dashed straight to the nearest shelter) and not to the escape strategy of rabbits which included zigzag runs, leaps, and back-tracks [30].

Nevertheless, the European rabbit has now been coexisting and interacting for some 150 years with the Chilean fauna and flora, so it is expected that it should be already embedded into the local ecosystems, building a network of interactions [52]. In fact, a sustained increase in the consumption of rabbits has been observed in recent times for the native eagle *Geranoaetus melanoleucus* and the native fox *Lycalopex culpaeus* [53,54,55,56]. For instance, *L. culpaeus* diet had 20% rabbit numerical frequency in 1976 [47], 37% in 1983 [53], and 48% in 1984 [57], all three studies conducted in the same locality. The increased rabbit intake paralleled the decrease in consumption of the native rodent *Octodon degus* and other small mammals due to the loss of shrub cover, fragmentation, and deterioration of their habitat [55]. Thus, rabbits may have currently become an important food resource for those native predators, especially in anthropized landscapes and dryland areas affected by drought [56], where populations of native small mammals have decreased.

On account of their high abundance and impacts, European rabbits should be controlled to protect Chilean biodiversity. Still, this species is possibly deeply involved in the local ecosystems, and any control effort could affect their communities, positively or negatively, producing undesired impacts on native endangered species. Therefore, understanding the ecological feedbacks that an eventual control of the rabbit could elicit in the ecosystem is key to designing its efficient management. In this way, it would be possible to identify those species that should be monitored during rabbit management to avoid potential undesired effects. Food-web models provide tools to better understand and predict the complex effects of an invasive species and the impact of their eradication or control in different ecosystems [58].

The knowledge of the rabbit food-web in Chile could provide insights on how an invasive species interacts with predators, herbivorous competitors, and food plants. A drastic decrease in rabbit abundance could elicit bottom-up impacts in predators with a restricted dietary range. Then, such predators could face a reduction in their abundance or shift their diet increasing predation pressure on native prey [59,60,61]. At the same time, as rabbits preferentially consume several plant species, a decrease in rabbit abundance could also produce top-down effects by herbivory release, with community and ecosystem effects depending on the plants’ characteristics (e.g., native or invasive). Hence, to analyze the rabbit food-web in a 36 yr monitored semi-arid ecosystem in Chile seems ideal to understand their interaction network, feedbacks with sympatric species, and their impacts on them, either positive or negative. We hypothesize that the European rabbit interacts directly and indirectly with several native and non-native species within the local food web. We are assessing whether rabbit persistence can positively impact predators by being a subsidy for them and negatively affect the plants through their consumption. It follows that eradicating the rabbit could negatively affect the persistence of predators, resulting in a reduction in their trophic niche (especially those with narrow dietary breadth), and positively impact the persistence of plants, because of their release from herbivory (especially on plants with fewer consumers).

## 2. Materials and Methods

### 2.1. Study Site

**Figure 1:**
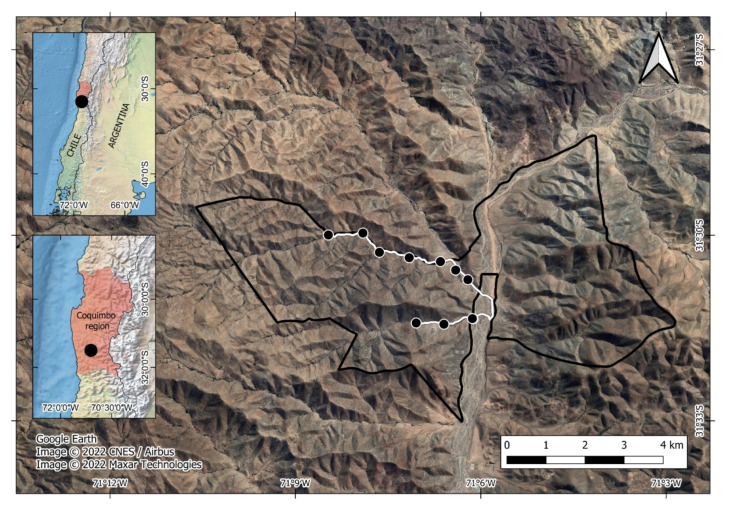
Study site at Las Chinchillas National Reserve in north-central Chile. Dots are sampling units for faunal data.

Las Chinchillas National Reserve is 17 km North of Illapel city, Coquimbo Region, north-central Chile (31°50′87″ S, 71°10′53″ W, Figure 1). It is a state-protected wilderness area, the only that harbors a sizable population of the native and endangered rodent *Chinchilla lanigera* [62,63]. The Reserve’s vegetation is typical of the semi-arid zone (Supplementary Material Table S1). The climate is characterized by cool winters (3 to 5 °C) with sporadic rains (150 mm) and dry summers with high temperatures (27 to 30 °C) [64].

The wildlife diversity of the Reserve comprises 80 vertebrate species, with birds being the most abundant and amphibians the least, with only one species [63,65] (Supplementary Material Table S2). Avian predators and scavengers, together with mammalian carnivores present in the Reserve are listed in Table 1. Their most common mammalian prey are listed in Table 2.

### 2.2. Bibliographic and Empirical Data

The bibliographic search to build the European rabbit food-web at the study site was based on an exhaustive review of dissertations, theses, scientific articles, book chapters, and technical reports published up to October 2022. The selection criteria required articles to indicate verifiable taxonomy, study site, diel activity, and body size, of animal and plant species present in the Reserve or nearby, and any interaction of rabbits with primary producers, competitors, and predators. We finally considered 95 selected documents that fulfilled the search requirements. We used a time series of faunal data and their diet from the Reserve to confirm the bibliographic consumer-resource relationship in the Reserve and build the corresponding food web, using the 36 years of monitoring fauna data of small mammals ([104] for more details), 22 years for rabbits and predators, and predators’ diet data. The dietary composition of each predator was obtained by analyzing feces (carnivores) and regurgitated pellets (raptors) collected across 17 km fixed transects each season, from 1987 to 2022. The feces and pellets were taken to the laboratory where they were gently disintegrated and analyzed, with prey consumed identified on the basis of bone, feather, hair, or scale remains found. The minimum number of prey present in the feces and pellets was estimated by the number of double or single anatomical elements of the prey present, such as the skulls, teeth rows, or jaws of mammals; the skulls, pelvises, or beaks of birds; the skulls, tails, or scales of reptiles; and the mandibles, stings, elytra, or wings of insects.

### 2.3. Food Web Construction

The food network was built based on the rabbit-centered interactions, identifying the predator species that consume local mammalian prey and the foods consumed by the latter in the Reserve. Then, the network was completed with an estimation of the rabbit as a potential competitor of native mammals for food within the Reserve. After this, through information obtained by bibliographic review and empirical data collection, a binary adjacency matrix ‘resource-consumer matrix’ was built with 0 s and 1 s. A value of 1 indicates the presence of trophic interaction, and 0 is the absence of a trophic interaction. Adjacency matrices were used to model the food web and to analyze its topology. Eight structural properties were calculated to analyze the food web topology. These were: species richness (S = number of species in the network); number of trophic links (L); link density (L/S); direct connectance (C = L/S^2^); proportion of basal, intermediate, and top species; standardized generality (nº prey/[L/S], henceforth, generality); and vulnerability (nº predators/[L/S], henceforth, vulnerability) [105]. These analyses were conducted using Network 3D software [106]. The rabbit’s relative importance within the food web was analyzed by ranking the number of links with which it was related, with the total number of links, ongoing links (number of prey), and outgoing links (number of predators) being thus calculated. In addition, the potential impact that rabbit management could have on the food web was analyzed by simulating the most extreme management scenario, i.e., rabbit eradication. Thus, the rabbit node was removed from the network, and then the top-down and bottom-up ecological mechanisms triggered by rabbit-node extinction were assessed. Trophic niche reduction was assessed as an index of the direct bottom-up effects, comparing the number of ongoing links of each species before and after rabbit eradication. Lastly, predation or herbivory release were assessed as an index of the direct top-down effects, comparing the number of outgoing links of each species before and after rabbit-node extinction. Frequency histograms were built to understand which ecological mechanisms predominated in the process of simulated rabbit removal. Histograms indicated the percentage of prey lost by predators and the percentage of prey released from herbivory pressure owing to the extinction of the rabbit node. These analyses were conducted in R-environment using the “igraph” and “network” packages [107].

## 3. Results

**Figure 2:**
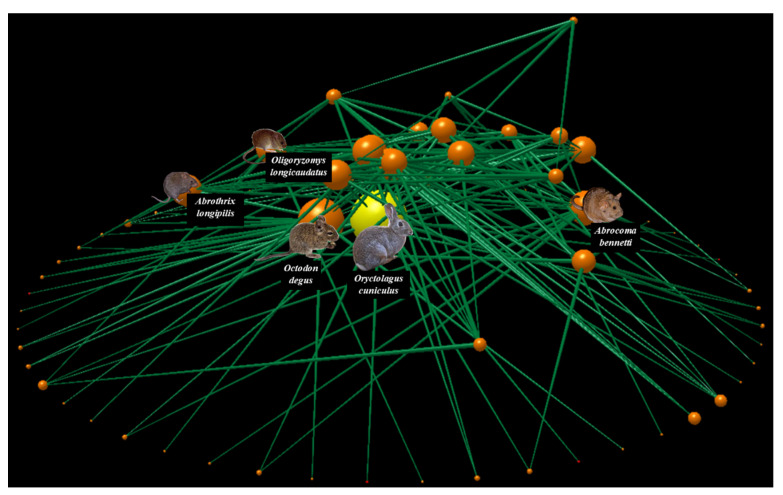
The food web of Las Chinchillas National Reserve in north-central Chile. The primary producers (plants), mid-level consumers (herbivores), and top-level consumers (predators) are shown from the bottom upwards. Nodes represent species and lines represent links of trophic interaction. Node size represents the number of total trophic interactions at which each node is associated. The yellow node represents the rabbit; orange nodes represent the remaining species. This figure was made using the Network3D program [106].

The food web of Las Chinchillas National Reserve (Figure 2, Supplementary Material Figure S1) was composed of 77 species, of which 69% were primary producers (plants), 18% were mid-level consumers or herbivores, and 13% were top-level consumers or predators. Of the 77 nodes, three corresponded to exotic species: European rabbits (*Oryctolagus cuniculus*) and the annual herbs squirrel tail fescue (*Vulpia bromoides*) and common stork’s-bill (*Erodium cicutarium*), representing 4% of the network. Additionally, 187 trophic interactions were documented, with a connectance of 0.03. On average, the nodes had a higher proportion of prey than of consumers, given that the values of generality and vulnerability were 1.8 and 1.2, respectively. The most connected species in the food web was the rabbit, followed by the native rodents degu (*Octodon degus*) and Bennett’s chinchilla rat (*Abrocoma bennetti*), with a connectivity of 5.6, 4.3, and 3.5, respectively. This means that the rabbit was the species that interacted with the greatest number of other species in the network (27 species, 35% of the network), most of them being native (e.g., the vertebrate predators *Lycalopex culpaeus*, *Parabuteo unicinctus*, and *Tyto alba*; the rodent herbivores *Abrocoma bennetti*, *Abrothrix longipilis*, and *Octodon degus*; the shrubs *Flourensia thurifera*, *Lithraea caustica*, and *Maytenus boaria*). Similarly, the rabbit was the species with the highest vulnerability (5.0) and generality (5.6) in the entire food web, indicating that, on average, the rabbit had more predators and food plants than the remaining species in the network. Therefore, it could be inferred that any intervention on rabbit abundance could either positively or negatively affect several native species.

**Figure 3:**
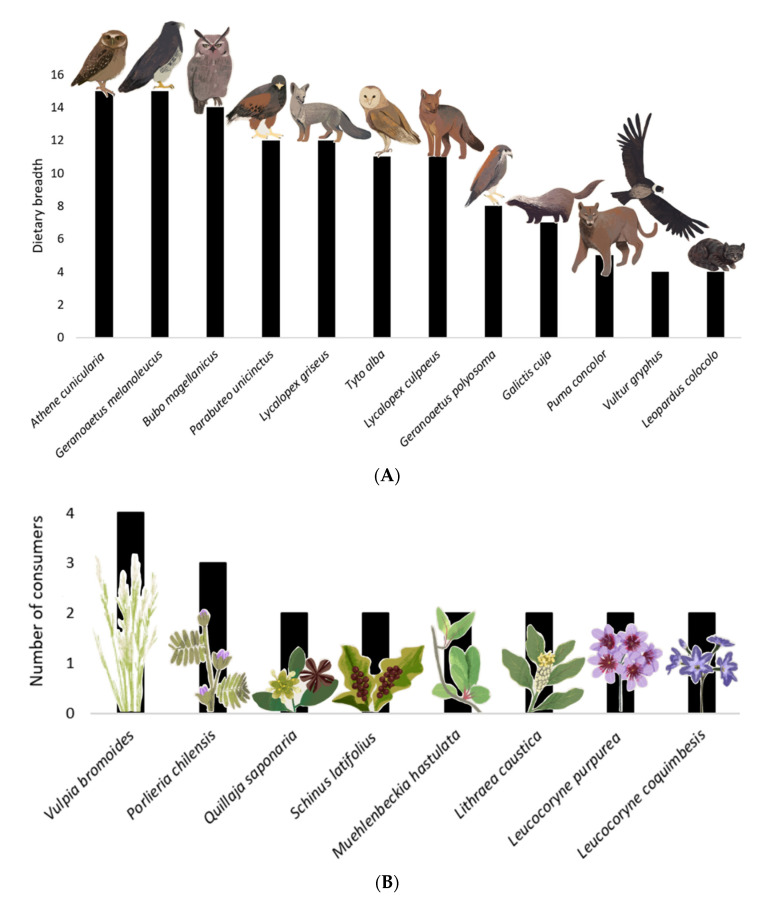
The relative importance of rabbit in the food web of Las Chinchillas National Reserve in north-central Chile. (**A**). Dietary breadth of rabbit predators, expressed as number of prey categories used. The diet composition of these predators is in Table 1. (**B**). Primary producers and the number of species consuming these food plants. These plants (and their consumers) were: *Vulpia bromoides* (*A. bennetti*, *A. olivaceus*, *O. degus*, and *O. cuniculus*); *Porlieria chilensis* (*L. culpaeus*, *O. degus* and *O. cuniculus*); *Lithraea caustica*, *Muehlenbeckia hastulata*, *Quillaja saponaria*, and *Schinus latifolius* (*O. degus* and *O. cuniculus*)*; Leucocoryne purpurea* and *Leucocoryne coquimbensis* (*O. cuniculus* and *S. cyanus*). Plants consumed only by rabbits (not represented in this Figure) were *Oxalis perdicaria*, *Plantago hispidula*, *Schizanthus parvulus*, *Senna cumminggi*, *Tropaeolum azureum*, *Flourensia thurifera*, and *Maytenus boaria*.

The European rabbit had 11 predator species (*Athene cunicularia*, *Bubo magellanicus*, *Galictis cuja*, *Geranoaetus melanoleucus*, *Geranoaetus polyosoma*, *Leopardus colocolo*, *Lycalopex culpaeus*, *Lycalopex griseus*, *Puma concolor*, *Parabuteo unicinctus*, and *Tyto alba*) and one scavenger species (*Vultur gryphus*); all of them native and with a dietary range of four to 15 prey species each (Figure 3A, Table 1). Therefore, the rabbit’s role as a prey or carrion resource is heterogeneous, and it is potentially more important for species with a lower dietary range in the Reserve. Three of the twelve rabbit consumers were among the most connected species in the network: the black-chested eagle (*Geranoaetus melanoleucus*), the burrowing owl (*Athene cunicularia*), and the Magellanic horned owl (*Bubo magellanicus*), which had the highest dietary ranges in the Reserve, and values of 6.2, 5.8, and 5.8 in generality, respectively. The remainder of the rabbit consumers had very low generality. For instance, the condor (*Vultur gryphus*) and pampas cat (*Leopardus colocolo*) had the same generality value of 1.65, and the puma (*Puma concolor*) had 2.1. Thus, these three predators could be negatively affected if the rabbit were eradicated.

Rabbits consumed a total of 15 food plants (Table 2, Figure 3B), with one of them being an exotic invasive herb, *Vulpia bromoides*, the rest being the native herbs *Leucocoryne purpurea*, *Leucocoryne coquimbensis*, *Oxalis perdicaria*, *Plantago hispidula*, *Schizanthus parvulus*, *Senna cumminggi*, and *Tropaeolum azureum*; and the native shrubs *Flourensia thurifera*, *Lithraea caustica*, *Maytenus boaria*, *Muehlenbeckia hastulata*, *Porlieria chilensis*, *Quillaja saponaria*, and *Schinus latifolius.* Then, if the rabbits are controlled or extirpated, this could relax their herbivory pressure and increase the plant biomass in the Reserve.

**Figure 4:**
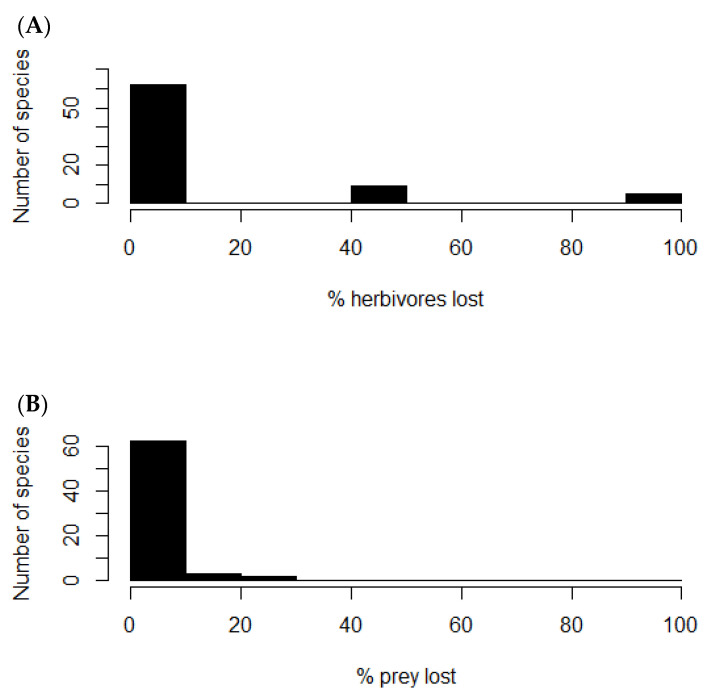
Frequency histograms. (**A**) Number of plant species versus percentage of herbivores lost after rabbit removal. (**B**) Number of predator species versus percentage of prey species lost after rabbit removal.

Removal of the rabbit node from the food web caused nine plants of the network to lose from 40 to 50% of their consumers (Figure 4A), which corresponded to the native shrubs *Flourensia thurifera*, *Lithraea caustica*, *Maytenus boaria*, *Muehlenbeckia hastulata*, *Porlieria chilensis*, *Quillaja saponaria*, and *Schinus latifolius;* and the native herbs *Leucocoryne coquimbesis* and *Leucocoryne purpurea.* Other five species of herbs lost from 90 to 100% of their consumers: *Oxalis perdicaria*, *Schizanthus parvulus*, *Plantago hispidula*, *Senna cumminggi*, and *Tropaeolum azureum*. On the other hand, twelve native predator species lost from 7 to 25% of their prey (Figure 4B), which corresponded to *Geranoaetus melanoleucus* (7%), *Athene cunicularia* (7%), *Bubo magellanicus* (7%), *Parabuteo unicinctus* (8%), *Lycalopex griseus* (8%), *Lycalopex culpaeus* (9%), *Tyto alba* (9%), *Geranoaetus polyosoma* (12%), *Galictis cuja* (14%), *Vultur gryphus* (20%), *Leopardus colocolo* (25%), and *Puma concolor* (25%). These results show how rabbit control or extirpation could trigger both top-down and bottom-up ecological feedbacks. Still, predation release may be predominant, even though the outcome of these mechanisms will depend on the strength of the interaction between the species involved, which is yet to be assessed.

## 4. Discussion

Invasive species are usually considered a threat to the conservation of diversity because they alter the structure and functioning of the invaded ecosystems [1]. The European rabbit has been coexisting and interacting with native and other invasive species in Chile for ca. 150 years, building complex interaction networks as a plant consumer, a competitor of other herbivores, as prey of predators, or as carrion for scavengers. Our results show that rabbits interact with several native and non-native species in Las Chinchillas National Reserve, allowing us to hypothesize the ecological feedback mechanisms that could operate if rabbits were controlled or eradicated and how this could propagate and affect the local food web [58,60,61].

The topology analysis of the Reserve’s food web shows that the rabbit is the most connected species therein and therefore is strongly embedded into the local community, playing a key role as prey of 12 avian and mammalian predators and scavengers, competitor of four rodents, and consumer of 14 species of herbs and shrubs. Thus, the rabbit major importance in the food web of the Reserve lies in its positive or negative impact on species with fewer interactions, which could be more vulnerable if rabbits were controlled or extirpated [59]. For instance, predators or scavengers with a narrow dietary range, such as *Galictis cuja*, *Geranoaetus polyosoma*, *Leopardus colocolo*, *Puma concolor*, and *Vultur gryphus* could be more dependent on rabbit abundance, in comparison to that of native rodents. Of these species, three are threatened: *Leopardus colocolo* is classified in the Near threatened conservation category by both the IUCN and the Ministry of the Environment (MMA) of Chile; *Vultur gryphus*, vulnerable according to IUCN and Near threatened according to MMA; and *Puma concolor*, which although under the IUCN global evaluation is in the Least Concern category, the national review by the MMA places it as Near threatened. Additionally, herbs and shrubs consumed mostly by rabbits, such as *Leucocoryne purpurea*, *Leucocoryne coquimbensis*, *Lithraea caustica*, *Muehlenbeckia hastulata*, *Quillaja saponaria*, and *Schinus latifolius* could increase their biomass if they were released from rabbit herbivory pressure by. Additionally, because rabbits may compete with the rodents *Octodon degus* and *Abrocoma bennetti* (the second and third most connected species in the local food web), after rabbit control they may gain increased interactions within the local food web. Thus, perturbations such as droughts on these rodents could destabilize the network through bottom-up effects [108].

Our results indicate two possible scenarios regarding how rabbit control could affect predators with a low dietary range in the Reserve. First, predators and scavengers dependent mainly on rabbits could decrease their local abundances due to lack of their main food source [60,61]. Then, those with greater mobility or wider home ranges may be able to move outside the Reserve to feed [61,109], but then become unprotected and in contact with anthropogenic risks such as hunting, poisoning, electrocution, collision with power lines, or waste consumption. The second scenario is that predators and scavengers may stay in the Reserve and intensify predation on alternative prey such as native rodents [61]. This may translate into more predation pressure on those populations, which currently suffer from an intense megadrought in central Chile [110,111]. Likewise, there could be cascading effects on scavengers that feed on rabbit predators (e.g., Andean condor) [93]. A case in point: Both in Argentina [112] and the USA [113] the puma population decreased because their main prey decreased in the first place. As a consequence, in Argentina, the puma expanded its dietary range by incorporating alternative prey [112], and in the USA, it reduced its individual body mass [113]. Although in Las Chinchillas National Reserve we have no empirical data on the consumption of rabbits by puma, in Río Cipreses National Reserve (O’Higgins Region of central Chile) it has been shown that rabbits constitute most of the puma diet [82], thus suggesting that puma at the Reserve may display similar responses as in Argentina or the USA.

Invasive species should be controlled because of their impacts on biodiversity and ecosystem functioning [114]. In Chile, rabbits have a large impact by their consumption of plant biomass, decreasing and fragmenting plant cover, affecting other species of fauna, and generating erosion [6,9,12,13,115]. Thus, it seems convenient to control them, but the positive impact that rabbits have as a food subsidy for native predators cannot be ignored. Interactions between predators with narrow dietary range and rabbits in the Reserve showed a strong relationship leading to predator extinction when the rabbit node was eliminated. Predators such *Galictis cuja*, *Geranoaetus polyosoma*, *Leopardus colocolo*, and *Puma concolor*, and the scavenger *Vultur gryphus*, lost from 12 to 25% of their prey categories. Hence, if the rabbit were to be controlled in the Reserve, it should be important to monitor predator and scavenger abundance trends by assessing their home range, their hunting for alternate prey, and their foraying outside the protected area, eventually leaving it.

Rabbits, in the absence or scarcity of predators, can become abundant enough to exert top-down pressure on local plants [23,116]. Hence, their herbivory is one of the main negative interactions that can hinder plant regeneration [16,117] and more so when their densities are high [17,24,118]. In addition, rabbits facilitate the establishment of invasive exotic plants, alter the habitat, and increase erosion [119,120,121]. Our results show that rabbit control could positively affect those plants mostly consumed by them, by relaxing the herbivory pressure and thus allowing plant regeneration [17,24,117,118,122]. Consequently, the biomass of those plants could increase, triggering bottom-up benefits for both native herbivores, predators, and scavengers. The benefits generated by rabbit control were experimentally shown by Holmgren et al. (2000) in a central Chilean site: When rabbits were excluded, native herbs increased strongly while the abundance of exotic herbs practically did not change. Further, rabbit herbivory favored the growth of prostrate herbs, which tended to be exotic, while rabbit exclusion favored erect herbs, which were native [123,124,125]. When rabbits were excluded, competition between native and exotic herbs decreased, and the biomass of the natives increased [123]. In light of this, the effect that rabbit control in the Reserve could have on the invasive *Vulpia bromoides* should be considered with caution, because the latter could increase in abundance and competitively harm native herb species. Indeed, studies of rabbit grazing in Europe indicate that they preferentially consume *V. bromoides*, thus being able to exert a positive impact by controlling this invasive species [126,127,128,129]. With regard to shrubs, rabbit control in the Reserve could increase the survival of species such as *Acacia caven*, *Baccharis linearis*, *Colliguaja odorifera*, *Peumus boldus*, *Prosopis chilensis*, and *Quillaja saponaria* [122,125,130]. In addition, it has been observed elsewhere in Chile that in the absence of herbivores, the sexual reproductive pathway is faster than the vegetative one for plant regrowth [131,132]. To witness, the eradication of rabbits from Chañaral and Choros land-bridge islands in northern Chile allowed a fast and visible recovery of the native vegetation [115,133]. Likewise, rabbits were eradicated from the oceanic Santa Clara Island off the Chilean coast from Valparaiso Region [115,120,134,135,136] and this allowed an important recovery of the native flora [121,136], highlighted by the reappearance of four endemic species [134]. In short, herbivory seems to be a limiting factor for Chilean plant species, and the exclusion of herbivores such as the rabbit may facilitate their regeneration. A worldwide meta-analysis by Barbar et al. (2016) reported that controlling rabbits may increase plant diversity almost immediately after removing only 30–40% of a local rabbit population [59]. However, in Chile this process does not seem to proceed that fast. For example, a long-term herbivore exclusion experiment with herbs in the semi-arid region of Chile obtained results that became evident only 20 years after its initiation [125]. Similarly, it took 34 years after an anti-herbivore exclusion was built in a temperate region of Chile, for the tree cover of an abandoned pasture to recover [137]. We admit that the European rabbit management that could be carried out in the Reserve is not the same as in an island eradication because of the larger area, more varied sources-sinks, and a complex trophic web where the rabbit is strongly embedded. Therefore, the importance of our results lies in recognizing the relevance of this invasive species in the Reserve’s trophic network and its possible cascading effects on predators and plants. The effects of adding physical barriers (plant protection, exclusion plots, and repellents) or introducing biological control agents (diseases or falconry), should be monitored for key species dependent on rabbits to evaluate the real impact of their removal.

Multispecies ecological network models provide wildlife managers with tools to understand and predict the complex effects of species removal or control in both intact and modified ecosystems [61]. Reduction or eradication of populations of invasive species can often lead to unexpected flow-on consequences for community structure and ecosystem processes if species interactions are not understood or accounted for by managers [138]. Our work highlights the idea that controlling a rabbit population in a semi-arid ecosystem is not a trivial pursuit because the rabbit decrease effect could be propagated either negatively or positively to other species in the network, causing diverse ecological feedbacks with cascading effects. It is important to consider that our results do not indicate that the same level of interaction should be found verbatim in other places that the rabbit has invaded. In fact, we suggest here to carry out similar studies of rabbit-centered food webs in different ecosystems to pinpoint the generality of our findings. In short, the food web approach provides information that is scientifically useful and complementary for the management of invasive species, because it provides a community-based perspective on how the impacts of management could spread to the species that are part of a given ecosystem. Finally, it should be noted that it is not enough to gauge only the relative importance of the rabbit (or any other mid-level consumer) in a given food web. Ideally, the absolute abundance of all consumer species should be obtained to evaluate the total consumption of prey by local predators, and estimate the dynamics of the food web through the use of bioenergetic approximations such as the allometric trophic network model and weighted networks with prey preference [139].

## Figures and Tables

**Table 1:** Alphabetic list of main predators and scavengers present in Las Chinchillas National Reserve in north-central Chile, their activity pattern, diet composition, and conservation status (using IUCN criteria).

Scientific Name	Common Name	Diel Activity	Diet Composition	Conservation Status	References
*Athene cunicularia*	Burrowing owl	Diurnal, Crepuscular	Mammals: *A. bennetti*, *A. longipilis*, *A. olivaceus*, *Chinchilla lanigera*, *O. degus*, *O. longicaudatus*, *O. cuniculus*, *S. cyanus*, *P. darwini*, *T. elegans.* Birds: Passerifomes. Reptiles: *Liolaemus fuscus.* Arthropods: Insects and arachnids, *Grammostola spathulate.*	Least Concern	[27,49,66,67,68]
*Bubo magellanicus*	Magellanic horned owl	Nocturnal	Mammals: *A. bennetti*, *A. longipilis*, *A. olivaceus*, *O. degus*, *O. lunatus*, *O. longicaudatus*, *O. cuniculus*, *P. darwini*, *S. cyanus*, *T. elegans.* Birds: *Nothoprocta perdicaria*, Passeriformes. Arthropods: Insects and arachnids.	Least Concern	[27,45,68,69,70]
*Galictis cuja*	Lesser grison	Diurnal	Mammals: *A. bennetti*, *A. longipilis*, *O. longicaudatus*, *O. cuniculus*, *P. darwini.* Reptiles: *Philodryas chamissonis.* Birds: Passeriformes.	Least Concern	[22,71,72]
*Geranoaetus melanoleucus*	Black-chested eagle	Diurnal	Mammals: *A. bennetti*, *A. longipilis*, *A. olivaceus*, *O. degus*, *O. longicaudatus*, *O. cuniculus*, *P. darwini*, *T. elegans.* Birds: *Colaptes pitius*, *Metriopelia melanoptera*, *Nothoprocta perdicaria*, *Schelorchilus albicollis*, Passeriformes. Reptiles: *Philodryas chamissonis*, Plus insects.	Least Concern	[54,73,74,75,76]
*Geranoaetus polyosoma*	Variable hawk	Diurnal	Mammals: *A. bennetti*, *A. olivaceus*, *O. degus*, *O. longicaudatus*, *P. darwini*, *O. cuniculus.* Reptiles: *Liolaemus platei.* Plus insects.	Least Concern	[48,68,77,78,79]
*Leopardus colocolo*	Pampas cat	Nocturnal	*O. cuniculus*, *P. darwini.* Plus birds and reptiles.	Near threatened	[71,80,81]
*Lycalopex culpaeus*	Culpeo fox	Nocturnal, Crepuscular	Mammals: *A. bennetti*, *A. longipilis*, *A. olivaceus*, *O. degus*, *O. longicaudatus*, *O. cuniculus*, *P. darwini*, *T. elegans.*, Reptiles: *Philodryas chamissonis.* Plants: *Porlieria chilensis.* Plus artropods.	Least Concern	[47,53,56,82,83,84,85]
*Lycalopex griseus*	Chilla fox	Continual	Mammals: *A. bennetti*, *A. longipilis*, *A. olivaceus*, *O. lunatus*, *O. degus*, *O. longicaudatus*, *O. cuniculus*, *P. darwini*, *T. elegans.* Reptiles: *Liolaemus nitidus*. Plus insects. Plants: *Ephedra andina.*	Least Concern	[47,71,86,87]
*Parabuteo unicinctus*	Harris’s hawk	Diurnal	Mammals: *A. bennetti*, *A. longipilis*, *A. olivaceus*, *O. longicaudatus*, *O. degus*, *O. cuniculus*, *P. darwini*, *S. cyanus*, *T. elegans.* Reptiles: *Philodryas chamissonis*, *Tachymenis chilensis*, *Pteroptochos megapodius.*	Least Concern	[27,47,55]
*Puma concolor*	Puma	Nocturnal, Crepuscular	Mammals: *A. longipilis*, *A. olivaceus*, *L. culpaeus*, *O. longicaudatus*, *O. cuniculus.*	Least Concern	[71,82,88]
*Tyto alba*	Barn owl	Nocturnal	Mammals: *A. bennetti*, *A. longipilis*, *A. olivaceus*, *O. lunatus*, *O. degus*, *O. longicaudatus*, *O. cuniculus*, *P. darwini*, *S. cyanus*, *T. elegans*. Birds: Passeriformes.	Least Concern	[46,86,89,90,91]
*Vultur gryphus*	Andean condor	Diurnal	Mammals: *Galictis cuja*, *Lycalopex culpaeus*, *O. cuniculus*, *Puma concolor.*	Vulnerable	[92,93]

**Table 2:** Alphabetic list of main mammalian prey present in Las Chinchillas National Reserve in north-central Chile, their activity pattern, diet composition, and conservation status (using IUCN criteria).

Scientific Name	Common Name	Diel Activity	Diet Composition	Conservation Status	References
*Abrocoma bennetti*	Bennett’s chinchilla rat	Nocturnal	Plants: *Acacia caven*, *Bridgesia incisifolia*, *Cordia decandra*, *Dioscorea humifusa*, *Ephedra andina*, *Flourensia thurifera*, *Nassella chilensis*, *Vulpia bromoides.*	Least Concern	[94]
*Abrothrix longipilis*	Long-haired grass mouse	Continual	Plants: *Alstroemeria diluta*, *Alstromeria angustifolia*, *Proustia baccharoides.* Arthropods and moss.	Least Concern	[94,95]
*Abrothrix olivaceus*	Olive grass mouse	Continual	Plants: *Acacia caven*, *Vulpia bromoides.* Plus insects and arachnids.	Least Concern	[96]
*Chinchilla lanigera*	Long-tailed chinchilla	Nocturnal	Plants: *Nassella chilensis*, *Adiantum chilense*, *Bridgesia incisifolia*, *Heliotropium stenophyllum*, *Lobelia polyphylla.*	Endangered	[97,98]
*Octodon degus*	Degu	Diurnal	Plants: *Baccharis linearis*, *Colliguaja odorifera*, *Erodium moschatum*, *Kageneckia oblonga*, *Lithraea caustica*, *Muehlenbeckia hastulata*, *Porlieria chilensis*, *Proustia cinérea*, *Quillaja saponaria*, *Schinus latifolius*, *Trevoa trinervis*, *Vulpia bromoides.*	Least Concern	[16,94,99,100,101,102]
*Octodon lunatus*	Moon-toothed degu	Crepuscular, Nocturnal	Plants: *Acacia caven*, and insects.	Near threatened	[103]
*Oligoryzomys longicaudatus*	Long-tailed rice mouse	Nocturnal	Plants: *Acacia caven*, *Chloraea* sp., *Erodium moschatum.* Arthropods and moss.	Least Concern	[89]
*Oryctolagus cuniculus*	European rabbit	Nocturnal	Plants: *Lithraea caustica*, *Quillaja saponaria*, *Schinus latifolius*, *Porlieria chilensis*, *Muehlenbeckia hastulata*, *Vulpia bromoides. Flourensia thurifera*, *Maytenus boaria*, *Leucocoryne coquimbesis*, *Leucocoryne purpurea*, *Oxalis perdicaria*, *Schizanthus parvulus*, *Plantago hispidula*, *Senna cumminggi*, *Tropaeolum azureum.*	Least Concern	[33]
*Phyllotis darwini*	Darwin’s leaf-eared mouse	Nocturnal	Plants: *Baccharis linearis*, *Erodium moschatum*, *Proustia cinerea.*	Least Concern	[94,99]
*Spalacopus cyanus*	Coruro	Diurnal	Plants: *Alstroemeria diluta*, *Alstromeria angustifolia*, *Dioscorea humifusa*, *Leuchocoryne coquimbesis*, *Leucocoryne purpurea*, *Sisyrinchium graminifolium*.	Least Concern	[94]
*Thylamys elegans*	Elegant mouse opossum	Nocturnal	Plants: *Erodium moschatum*, *Lycium chilensis*. Plus insects and arachnids.	Least Concern	[94,99]

## Data Availability

The data is unavailable due to privacy statement.

## Supplementary Materials

The following supporting information can be downloaded at: https://www.mdpi.com/article/10.3390/life13040916/s1, Table S1: Plants in Las Chinchillas National Reserve, Table S2: Amphibians, arthropods, birds, mammals, and reptiles in Las Chinchillas National Reserve, Table S3: Records of prey in *Athene cunicularia* diet between 1973 and 2004, in percentage of biomass of their prey, based on pellet analyses made in different studies throughout time, Table S4: Records of prey in the *Bubo magellanicus* diet between 1973 and 2001, in percentage of biomass of their prey, based on pellet analyses made in different studies throughout time, Table S5: Records of prey in the *Geranoaetus melanoleucus* diet between 1973 and 1989, in percentage of biomass of their prey, based on pellet analyses made in different studies throughout time, Table S6: Records of prey in *Lycalopex culpaeus* diet between 1973 and 2015, in percentage of biomass of their prey, based on feces analyses made in different studies throughout time, Table S7: Records of prey in *Lycalopex griseus* diet between 1973 and 2001, in percentage of biomass of their prey, based on feces analyses made in different studies throughout time, Table S8: Records of prey in the *Parabuteo unicinctus* diet, in percentage of biomass of their prey, based on pellet analyses made in different studies throughout time, Table S9: Records of prey in the *Tyto alba* diet between 1974 and 2001, in percentage of biomass of their prey, based on pellet analyses made in different studies throughout time, Figure S1: Predators, prey, and competitors in Las Chinchillas National food web [45,140,141,142,143,144,145,146,147,148,149,150,151,152,153,154,155,156,157,158,159,160,161,162,163,164,165,166,167,168,169,170,171,172,173,174,175,176,177].

## References

[1] Jaksic F.M., Castro S.A. (2021). Biological Invasions in the South American Anthropocene.

[2] WWF (2020). Living Planet Report 2020—Bending the Curve of Biodiversity Loss.

[3] Boudouresque C., Ruitton S., Verlaque M., Velikova V., Chipev N. (2005). Large-Scale Disturbances, Regime Shift and Recovery in Littoral Systems Subject to Biological Invasions. Large-Scale Disturbances (Regime Shifts) and Recovery in Aquatic Ecosystems: Challenges for Management Towards Sustainability.

[4] Vitousek P.M., D’Antonio C.M., Loope L.L., Rejmánek M., Westbrooks R. (1997). Introduced Species: A Significant Component of Human-Caused Global Change. N. Z. J. Ecol..

[5] Aronson J. (1992). Evolutionary Biology of Acacia Caven (Leguminosae, Mimosoideae): Infraspecific Variation in Fruit and Seed Characters. Ann. Mo. Bot. Gard..

[6] Camus P., Castro S., Jaksic F. (2008). El Conejo Europeo En Chile: Historia de Una Invasión Biológica. Historia.

[7] Luque G.M., Bellard C., Bertelsmeier C., Bonnaud E., Genovesi P., Simberloff D., Courchamp F. (2014). The 100^th^ of the World’s Worst Invasive Alien Species. Biol. Invasions.

[8] Correa-Cuadros J.P., Flores-Benner G., Muñoz-Rodríguez M.A., Briceño C., Díaz M., Strive T., Vásquez F., Jaksic F.M. (2022). History, Control, Epidemiology, Ecology, and Economy of the Invasion of European Rabbits in Chile: A Comparison with Australia. Biol. Invasions.

[9] Jaksic F.M. (1998). Vertebrate Invaders and Their Ecological Impacts in Chile. Biodivers. Conserv..

[10] Jaksic F.M., Iriarte J.A., Jiménez J.E., Martínez D.R. (2002). Invaders Without Frontiers: Cross-Border Invasions of Exotic Mammals. Biol. Invasions.

[11] Jaksic F., Yáñez J. (1983). Rabbit and Fox Introductions in Tierra Del Fuego: History and Assessment of the Attempts at Biological Control of the Rabbit Infestation. Biol. Conserv..

[12] Camus P., Castro S., Jaksic F. (2014). Reconstrucción Histórica de La Invasión Del Conejo Europeo (*Oryctolagus cuniculus*) En Chile Central: Lecciones Para Un Mejor Diálogo Entre Científicos y Gestores. Libro Invasiones Biológicas en Chile: Causas Globales e Impactos Locales.

[13] Camus P., Castro S.A., Jaksic F.M., Jaksic F.M., Castro S.A. (2021). European Rabbit (*Oryctolagus cuniculus* L.) in Chile: The Human Dimension Behind a Biological Invasion. Biological Invasions in the South American Anthropocene: Global Causes and Local Impacts.

[14] Fuentes E.R., Simonetti J.A. (1982). Plant Patterning in the Chilean Matorral: Are the Roles of Native and Exotic Mammals Different?.

[15] Simonetti J.A., Fuentes E.R. (1983). Shrub Preferences of Native and Introduced Chilean Matorral Herbivores. Acta. Oecol. Oecol. Appl..

[16] Fuentes E.R., Jaksić F.M., Simonetti J.A. (1983). European Rabbits versus Native Rodents in Central Chile: Effects on Shrub Seedlings. Oecologia.

[17] Fuentes E., Hoffmann A., Poiani A., Alliende M. Vegetation Change in Large Clearings: Patterns in the Chilean Matorral | SpringerLink. https://link.springer.com/article/10.1007/BF01036739.

[18] Cuevas J.G., Van Leersum G. (2001). Project “Conservation, Restoration, and Development of the Juan Fernández Islands, Chile”. Rev. Chil. Hist. Nat..

[19] Fernández A., Sáiz F. (2007). The European Rabbit (*Oryctolagus cuniculus* L.) as Seed Disperser of the Invasive Opium Poppy (*Papaver somniferum* L.) in Robinson Crusoe Island, Chile. Mastozool. Neotrop..

[20] Jaksic F.M., Fuentes E.R., Yañez J.L. (1979). Spatial Distribution of the Old World Rabbit (*Oryctolagus cuniculus*) in Central Chile. J. Mammal..

[21] Jaksic F.M., Fuentes E.R. (1988). El Conejo Español: ¿Un Convidado de Piedra?. Ecología del Paisaje en Chile Central: Estudios sobre Sus Espacios Montañosos.

[22] Jaksic F.M., Fuentes E.R. (1991). Ecology of a Successful Invader: The European Rabbit in Central Chile. Biogeography of Mediterranean Invasions.

[23] Jaksic F.M., Fuentes E.R. (1980). Why Are Native Herbs in the Chilean Matorral More Abundant Beneath Bushes: Microclimate or Grazing?. J. Ecol..

[24] Holmgren M., Avilés R., Sierralta L., Segura A.M., Fuentes E.R. (2000). Why Have European Herbs so Successfully Invaded the Chilean Matorral? Effects of Herbivory, Soil Nutrients, and Fire. J. Arid Environ..

[25] Gálvez-Bravo L., Salvador A., Barja I. (2017). Conejo *Oryctolagus cuniculus* (Linnaeus, 1758). Enciclopedia Virtual de los Vertebrados Españoles.

[26] Myers K., Parer I., Wood D., Cooke B.D., Thompson K., King C.M. (1994). The Rabbit in Australia. The European Rabbit. The History and Biology of a Successful Colonizer.

[27] Jaksic F.M., Greene H.W., Yáñez J.L. (1981). The Guild Structure of a Community of Predatory Vertebrates in Central Chile. Oecologia.

[28] Jaksic F.M., Soriguer R.C. (1981). Predation Upon the European Rabbit (*Oryctolagus cuniculus*) in Mediterranean Habitats of Chile and Spain: A Comparative Analysis. J. Anim. Ecol..

[29] Jaksic F., Ostfeld R.S. (1983). Numerical and Behavioral Estimates of Predation upon Rabbits in Mediterranean-Type Shrublands: A Paradoxical Case. Rev. Chil. Hist. Nat..

[30] Jaksic F.M. (1986). Predation upon Small Mammals in Shrublands and Grasslands of Southern South America: Ecological Correlates and Presumable Consequences. Rev. Chil. Hist. Nat..

[31] Newsome A.E., Parer I., Catling P.C. (1989). Prolonged Prey Suppression by Carnivores—Predator-Removal Experiments. Oecologia.

[32] Villafuerte R. (1994). Riesgo de Predación y Estrategias Defensivas Del Conejo, Oryctolagus cuniculus, en El Parque Nacional de Doñana.

[33] Villafuerte R., Delibes-Mateo M., Palomo J., Gisbert J., Blanco J.C. (2007). Conejo—*Oryctolagus cuniculus* (Linnaeus, 1758). Atlas y Libro Rojo de los Mamíferos Terrestres de España.

[34] Hanski I., Henttonen H., Korpimäki E., Oksanen L., Turchin P. (2001). Small-Rodent Dynamics and Predation. Ecology.

[35] Sinclair A.R.E. (2003). Mammal Population Regulation, Keystone Processes and Ecosystem Dynamics. Philos. Trans. R. Soc. Lond. Ser. B Biol. Sci..

[36] Myers K. (1964). Influence of Density on Fecundity, Growth Rates, and Mortality in the Wild Rabbit. CSIRO Wildl. Res..

[37] Jaksic F., Fuentes E.R., Yáñez J. (1979). Two Types of Adaptation of Vertebrate Predators to Their Prey. Arch. Biol. Med. Exp..

[38] Trout R.C., Tittensor A.M. (1989). Can Predators Regulate Wild Rabbit *Oryctolagus cuniculus* Population Density in England and Wales?. Mammal Rev..

[39] Hansson L. (1971). Small Rodent Food, Feeding and Population Dynamics: A Comparison between Granivorous and Herbivorous Species in Scandinavia. Oikos.

[40] Hansson L. (1979). Food as a Limiting Factor for Small Rodent Numbers. Oecologia.

[41] Hansson L. (1987). An Interpretation of Rodent Dynamics as Due to Trophic Interactions. Oikos.

[42] Berryman A.A. (1999). Principles of Population Dynamics and Their Application.

[43] Hawkins B.A., Cornell H.V. (2008). Theoretical Approaches to Biological Control.

[44] Calvete C. (1999). Epidemiología de Enfermedad Hemorrágica (VHD) y Mixomatosis en El Conejo Silvestre (Oryctolagus cuniculus L. 1758) en El Valle Medio Del Ebro: Modelización de VHD y Herramientas de Gestión.

[45] Yañez J., Rau J., Jaksic F. (1978). Estudio comparativo de la alimentación de *Bubo virginianus* (Strigidae) en dos regiones de Chile. An. Mus. Hist. Nat. Valparaíso.

[46] Jaksic F.M., Yáñez J.L. (1979). The Diet of the Barn Owl in Central Chile and Its Relation to the Availability of Prey. Auk.

[47] Jaksic F.M., Schlatter R.P., Yáñez J.L. (1980). Feeding Ecology of Central Chilean Foxes, *Dusicyon culpaeus* and *Dusicyon griseus*. J. Mammal..

[48] Jaksic F.M., Yáñez J.L., Schlatter R.P. (1980). Prey of the Harris’ Hawk in Central Chile. Auk.

[49] Schlatter R.P., Yáñez J.L., Núñez H., Jaksić F.M. (1980). The Diet of the Burrowing Owl in Central Chile and Its Relation to Prey Size. Auk.

[50] Schlatter R.P., Yáñez J.L., Jaksić F.M. (1980). Food-Niche Relationships between Chilean Eagles and Red-Backed Buzzards in Central Chile. Auk.

[51] Jaksic F., Yáñez J. (1980). ¿Quién controla las poblaciones de conejos introducidos?. Medio Ambiente.

[52] Dunne J., Pascual M., Dunne J. (2006). The Network Structure of Food Webs. Ecological Networks: Linking Structure to Dynamics in Food Webs.

[53] Simonetti J. (1986). Human-Induced Dietary Shift in *Dusicyon culpaeus*. Mammalia.

[54] Pavez E., Jiménez J. (1992). Diet Shifts of Black-Chested Eagles (*Geranoaetus melanoleucus*) from Native Prey to European Rabbits in Chile. J. Raptor Res..

[55] Pavez E.F., Lobos G.A., Jaksic F.M. (2010). Long-Term Changes in Landscape and in Small Mammal and Raptor Assemblages in Central Chile. Rev. Chil. Hist. Nat..

[56] Rubio A., Alvarado R., Bonacic C. (2013). Introduced European Rabbit as Main Prey of the Native Carnivore Culpeo Fox (*Lycalopex culpaeus*) in Disturbed Ecosystems of Central Chile: *Stud*. Neotrop. Fauna Environ..

[57] Iriarte J.A., Jimenez J.E., Contreras L.C., Jaksić F.M. (1989). Small-Mammal Availability and Consumption by the Fox, *Dusicyon culpaeus*, in Central Chilean Scrublands. J. Mammal..

[58] Buenavista S., Palomares F. (2018). The Role of Exotic Mammals in the Diet of Native Carnivores from South America. Mammal Rev..

[59] Barbar F., Hiraldo F., Lambertucci S.A. (2016). Medium-Sized Exotic Prey Create Novel Food Webs: The Case of Predators and Scavengers Consuming Lagomorphs. PeerJ.

[60] Barbar F., Lambertucci S.A. (2018). The Roles of Leporid Species That Have Been Translocated: A Review of Their Ecosystem Effects as Native and Exotic Species. Mammal Rev..

[61] Lurgi M., Ritchie E.G., Fordham D.A. (2018). Eradicating Abundant Invasive Prey Could Cause Unexpected and Varied Biodiversity Outcomes: The Importance of Multispecies Interactions. J. Appl. Ecol..

[62] Prado J.A. (1996). Plan de Manejo Reserva Nacional Las Chinchillas..

[63] Piñones C., Povea P., Silva J. (2011). Ruta Naturalista Reserva Nacional Las Chinchillas. La Chiricoca.

[64] Weather Spark El Clima en ILLAPEL, El Tiempo Por Mes, Temperatura Promedio (Chile). https://es.weatherspark.com/y/25820/Clima-promedio-en-Illapel-Chile-durante-todo-el-a%C3%B1o#Sections-Precipitation.

[65] MMA M., del M.A. Listado de Especies Clasificadas Desde El 1° al 17° Proceso de Clasificación RCE (Actualizado a Mayo de 2022). https://clasificacionespecies.mma.gob.cl/.

[66] Torres-Contreras H., Silva-Aranguiz E., Jaksic F.M. (1994). Dieta y selectividad de presas de *Speotyto cunicularia* en una localidad semi-árida del norte de Chile a lo largo de siete años (1987–1993). Rev. Chil. Hist. Nat..

[67] Cruz-Jofré F., Vilina Y.A. (2014). Ecología Trófica de *Athene cunicularia* (Aves: Strigidae) En Un Sistema Insular Del Norte de Chile: ¿Posible respuesta funcional y numérica frente a *Pelecanoides garnotii* (Aves: Pelecanoididae)?. Gayana Concepc..

[68] Figueroa R., Alvarado Orellana S.A., Corales E., González-Acuña D., Schlatter R., Martinez D.R. (2015). Búhos de Chile. Los Búhos Neotropicales: Diversidad y Conservación.

[69] Mella J.E. (2002). Dieta Del Cernícalo (*Falco sparverius*) y Del Tucúquere (*Bubo magellanicus*) En Un Ambiente Cordillerano de Chile Central. Bol. Chil. Ornitol..

[70] Muñoz-Pedreros A., Yáñez J., Gil C., Norambuena H.V., Carmona E.R. (2017). Spatial Differences in the Diet of the Magellanic Horned Owl *Bubo magellanicus* (Gmelin, 1788) in Central Chile. N. Z. J. Zool..

[71] Iriarte A., Jaksic F. (2012). Los Carnívoros de Chile.

[72] Ferreira J.L.P., Rodrigues N.L.A., Silva C.M.A., Uchôa J.S., dos Santos F.G.P., Barroso de Andrade E. (2022). Primeiro registro documentado do furão-pequeno *Galictis cuja* (Molina, 1782) no estado do Piauí, Nordeste do Brasil. Pesqui. Ensino Ciênc. Exatas Nat..

[73] Galende G.I., Trejo A. (2003). Depredación del águila mora (*Geranoaetus melanoleucus*) y el búho (*Bubo magellanicus*) sobre el chinchillón (*Lagidium viscacia*) en dos colonias del noroeste de Patagonia, Argentina. Mastozool. Neotrop. J..

[74] Jiménez J.E., Jaksić F.M. (1989). Behavioral Ecology of Grey Eagle-Buzzards, *Geranoaetus melanoleucus*, in Central Chile. Condor.

[75] Jiménez J.E., Jaksic F.M. (1990). Historia natural del águila (*Geranoaetus melanoleucus*): Una revisión. El Hornero.

[76] Trejo A., Kun M., Seijas S. (2006). Dieta Del Águila Mora (*Geranoaetus melanoleucus*) En Una Transecta Oeste-Este En El Ecotono Norpatagónico. El Hornero.

[77] Valladares P., Álvarez Henríquez N., Urrutia Osorio N., Olivares Zuleta F., Alvarado Orellana S. (2015). Dieta Del Aguilucho Común *Geranoaetus polyosoma* (Quoy & Gaimard 1824) En La Región de Atacama, Chile. Gayana Concepc..

[78] Dellacasa V. (2005). Estudio de Los Tipos de Vuelo Del Aguilicho Común (Buteo polyosoma) Durante El Período Estival En Nevados de Chillán, Centro-Sur de Chile.

[79] Jiménez J.E. (1995). Historia Natural Del Aguilucho *Buteo polyosoma*: Una Revisión. Hornero.

[80] Tellaeche C.G. (2015). Ecología y Uso del Espacio de Dos Especies de Félidos, Gato Andino (Leopardus jacobita) y Gato del Pajonal (L. colocolo) en La Región Altoandina, Provincia de Jujuy.

[81] Huaranca J.C., Villalba M.L., Negrões N., Jiménez J.E., Macdonald D.W., Pacheco L.F., Huaranca J.C., Villalba M.L., Negrões N., Jiménez J.E. (2019). Density and Activity Patterns of Andean Cat and Pampas Cat (*Leopardus jacobita* and *L. colocolo*) in the Bolivian Altiplano. Wildl. Res..

[82] Osorio C., Muñoz A., Guarda N., Bonacic C., Kelly M. (2020). Exotic Prey Facilitate Coexistence between Pumas and Culpeo Foxes in the Andes of Central Chile. Diversity.

[83] Zúñiga A.H., Fuenzalida V. (2016). Dieta Del Zorro Culpeo (*Lycalopex culpaeus* Molina 1782) En Un Área Protegida Del Sur de Chile. Mastozool. Neotrop..

[84] Yañez J., Jaksic F. (1978). Rol Ecológico de Los Zorros (Dusicyon) En Chile Central. An. Mus. Hist. Nat. Valparaíso.

[85] Ebensperger L.A., Mella J.E., Simonetti J.A. (1991). Trophic-Niche Relationships among *Galictis cuja*, *Dusicyon culpaeus*, and *Tyto alba* in Central Chile. J. Mammal..

[86] Correa P., Roa A. (2005). Relaciones Tróficas Entre *Oncifelis guigna*, *Lycalopex culpaeus*, *Lycalopex griseus* y *Tyto alba* En Un Ambiente Fragmentado de La Zona Central de Chile. Mastozool. Neotrop..

[87] Muñoz-Pedreros A., Yáñez J., Norambuena H.V., Zúñiga A. (2018). Diet, Dietary Selectivity and Density of South American Grey Fox, *Lycalopex griseus*, in Central Chile. Integr. Zool..

[88] Rau J., Jiménez J.E. (2002). Diet of Puma (*Puma concolor*, Carnivora: Felidae) in Coastal and Andean Ranges of Southern Chile. Stud. Neotrop. Fauna Environ..

[89] González D., Ausset Salgado M., Skewes Ramm O., Figueroa Rojas R.A. (2004). Variación Estacional En El Consumo de Roedores Por La Lechuza de Campanario (*Tyto alba*) En Un Área Suburbana de Chillán, Centro-Sur de Chile. El Hornero.

[90] Muñoz-Pedreros A., Gil C., Yáñez J., Rau J.R., Möller P. (2016). Trophic Ecology of Two Raptors, Barn Owl (*Tyto alba*) and White-Tailed Kite (*Elanus leucurus*), and Possible Implications for Biological Control of Hantavirus Reservoir in Chile. Wilson J. Ornithol..

[91] Zurita C., Erazo A., Opitz M., Volosky T. (2018). Sobreposición de dieta estacional de tucúquere (*Bubo magellanicus*) y lechuza blanca (*Tyto alba*) mediante el estudio de egagrópilas en el parque nacional río clarillo. Biodiversidata Conserv. Gest. Manejo Áreas Silv..

[92] Pavez E.F. (2014). Patrón de movimiento de dos cóndores andinos *Vultur gryphus* (Aves: Cathartidae) en los andes centrales de chile y argentina. Bol. Chil. Ornitol..

[93] Duclos M., Sabat P., Newsome S.D., Pavez E.F., Galbán-Malagón C., Jaksic F.M., Quirici V. (2020). Latitudinal Patterns in the Diet of Andean Condor (*Vultur gryphus*) in Chile: Contrasting Environments Influencing Feeding Behavior. Sci. Total Environ..

[94] Iriarte W. (2008). Mamíferos de Chile.

[95] Polop F., Sepúlveda L., Sbriller A.P., Polop J., Provensal M.C. (2014). Food Habits of *Oligoryzomys longicaudatus* (Rodentia) in a Steppe–Forest Transitional Area of Argentinean Patagonia. Ecol. Austral.

[96] Spotorno O. A.E., Palma V. R.E., Valladares F. J.P. (2000). Biología de Roedores Reservorios de Hantavirus En Chile. Rev. Chil. Infectol..

[97] Spotorno A.E., Zuleta C.A., Valladares J.P., Deane A.L., Jiménez J.E. (2004). Chinchilla Laniger. Mamm. Species.

[98] Valladares Faúndez P., Spotorno Oyarzún Á., Zuleta Ramos C. (2014). Natural History of the Chinchilla Genus (Bennett 1829): Considerations of Their Ecology, Taxonomy and Conservation Status. Gayana Concepc..

[99] Meserve P.L. (1981). Trophic Relationships among Small Mammals in a Chilean Semiarid Thorn Scrub Community. J. Mammal..

[100] Meserve P.L., Martin R.E., Rodriguez J. (1984). Comparative Ecology of the Caviomorph Rodent *Octodon degus* in Two Chilean Mediterranean-Type Communities. Rev. Chil. Hist. Nat..

[101] Zunino S., Saiz F. (1991). Estructura y Densidad Poblacional de *Octodon degus* Mol. Stud. Neotrop. Fauna Environ..

[102] Bozinovic F. (1995). Nutritional Energetics and Digestive Responses of an Herbivorous Rodent (*Octodon degus*) to Different Levels of Dietary Fiber. J. Mammal..

[103] Gallardo M., Ojeda R.A., González C., Ríos C. (2007). The Octodontidae Revisited.

[104] Lima M., Stenseth N.C., Leirs H., Jaksic F.M. (2003). Population Dynamics of Small Mammals in Semi-Arid Regions: A Comparative Study of Demographic Variability in Two Rodent Species. Proc. R. Soc. Lond. B Biol. Sci..

[105] de Santana C.N., Rozenfeld A.F., Marquet P.A., Duarte C.M. (2013). Topological Properties of Polar Food Webs. Mar. Ecol. Prog. Ser..

[106] Williams R. (2010). Visualizing and Modelling Food Webs and Other Complex Networks.

[107] Csardi G., Nepusz T. The Igraph Software Package for Complex Network Research. https://igraph.org/.

[108] Courchamp F., Chapuis J.-L., Pascal M. (2003). Mammal Invaders on Islands: Impact, Control and Control Impact. Biol. Rev..

[109] Infante J. (2019). New Interactions in a Mammalian Community: Introduced Lagomorphs Sustain Native Carnivores in the Andes of Central Chile.

[110] Garreaud R.D., Boisier J.P., Rondanelli R., Montecinos A., Sepúlveda H.H., Veloso-Aguila D. (2019). The Central Chile Mega Drought (2010–2018): A Climate Dynamics Perspective. Int. J. Climatol..

[111] Lobos G., Tapia G., Alzamora A., Rebolledo N., Salinas H., Trujillos J.C., Girón G., Ascanio R. (2020). Dieta del zorro culpeo *Lycalopex culpaeus* (molina, 1782) durante la megasequía de chile central: Rol del ganado y evidencia de una alta interacción trófica entre mamíferos carnívoros. Mastozool. Neotrop..

[112] Branch L.C., Pessino M., Villarreal D. (1996). Response of Pumas to a Population Decline of the Plains Vizcacha. J. Mammal..

[113] Laundré J.W., Hernández L., Clark S.G. (2007). Numerical and Demographic Responses of Pumas to Changes in Prey Abundance: Testing Current Predictions. J. Wildl. Manag..

[114] Simberloff D., Martin J.-L., Genovesi P., Maris V., Wardle D.A., Aronson J., Courchamp F., Galil B., García-Berthou E., Pascal M. (2013). Impacts of Biological Invasions: What’s What and the Way Forward. Trends Ecol. Evol..

[115] Programa de las Naciones Unidas para el Desarrollo (PNUD) (2017). Valoración Económica Del Impacto de 7 Especies Exóticas Invasoras a Los Sectores Productivos y Biodiversidad En Chile: Estimaciones Preliminares.

[116] Zavaleta E.S., Hobbs R.J., Mooney H.A. (2001). Viewing Invasive Species Removal in a Whole-Ecosystem Context. Trends Ecol. Evol..

[117] Padilla F.M., Pugnaire F.I. (2006). The Role of Nurse Plants in the Restoration of Degraded Environments. Front. Ecol. Environ..

[118] Cuevas J.G., Quesne C.L. (2006). Low Vegetation Recovery after Short-Term Cattle Exclusion on Robinson Crusoe Island. Plant Ecol..

[119] CONAF (1998). Plan de Manejo Parque Nacional Archipiélago de Juan Fernández.

[120] CONAF (2009). Plan de Manejo Parque Nacional Archipiélago Juan Fernández.

[121] Penneckamp D. (2018). Flora Vascular Silvestre Del Archipiélago Juan Fernández.

[122] Cuevas J.G., Silva S.I., León-Lobos P., Ginocchio R. (2013). El Efecto Nodriza y La Exclusión de Herbivoría Facilitan La Colonización de Plantas En Depósitos de Relaves Mineros Abandonados En Chile Centro-Norte. Rev. Chil. Hist. Nat..

[123] Holmgren M., Segura A.M., Fuentes E.R. (2000). Limiting Mechanisms in the Regeneration of the Chilean Matorral—Experiments on Seedling Establishment in Burned and Cleared Mesic Sites. Plant Ecol..

[124] Manrique R., Gutiérrez J.R., Holmgren M., Squeo F.A. (2007). Reduced Herbivory during Simulated ENSO Rainy Events Increases Native Herbaceous Plants in Semiarid Chile. Plant Ecol..

[125] Gutiérrez J.R., Meserve P.L., Kelt D.A., Engilis A., Previtali M.A., Milstead W.B., Jaksic F.M. (2010). Long-Term Research in Bosque Fray Jorge National Park: Twenty Years Studying the Role of Biotic and Abiotic Factors in a Chilean Semiarid Scrubland. Rev. Chil. Hist. Nat..

[126] Diaz I. (1999). Food Habits of the Rufous-Legged Owl (*Strix rufipes*) in the Mediterranean Sclerophyllous Forest of Central Chile. J. Raptor Res..

[127] Boorman L.A., Fuller R.M. (1982). Effects of Added Nutrients on Dune Swards Grazed by Rabbits. J. Ecol..

[128] Crawley M.J. (1990). Rabbit Grazing, Plant Competition and Seedling Recruitment in Acid Grassland. J. Appl. Ecol..

[129] ten Harkel Matthijs J., van der Meulen F. (1996). Impact of Grazing and Atmospheric Nitrogen Deposition on the Vegetation of Dry Coastal Dune Grasslands. J. Veg. Sci..

[130] Droguett P.V. (2019). Efecto de La Exclusión de Hervíboros, Incendios y Tipo de Parche de Vegetación sobre La Regeneración Natural de La Vegetación Leñosa de Chile Central.

[131] Becerra P., Smith-Ramírez C., Arellano E. (2019). Evaluación de Técnicas Pasivas y Activas Para La Recuperación del Bosque Esclerófilo de Chile Central.

[132] Gómez N. (2020). Facilitation by Pioneer Trees and Herbivore Exclusion Allow Regeneration and Succession of Woody Species in a Semiarid Ecosystem.

[133] Island Conservation (2014). Restauración Ecológica de La Reserva Nacional Pingüino de Humboldt: Erradicación de Conejo Europeo de La Isla Choros.

[134] Ojeda P., González H., Araya G. (2003). Erradicación Del Conejo Europeo Oryctolagus cuniculus Linnaeus 1758 Desde La Isla Santa Clara Archipiélago de Juan Fernández.

[135] Sáez F., Araya G., Meza J., Leiva I., Quilaqueo R. (2019). Evolución de La Restauración En Isla Santa Clara Posterior a La Erradicación de Mamíferos Exóticos Invasores Restoration Evolution at Santa Clara Island after the Eradication of Invasive Exotic Mammals. Conserv. Gest. Manejo Áreas Silv. Protegidas.

[136] Saunders G., Glen A., Campbell K., Atkinson R., Sawyer J., Hagen E., Torres H. (2011). Estudio Sobre La Factibilidad Del Manejo de Especies Invasoras Em El Archipiélago de Juan Fernández, Chile.

[137] Montaldo B. (1999). Treinta y Cuatro Años de Una Sucesión Secundaria En Pradera de Ñadi En La Provincia de Valdivia, Chile. Agro Sur.

[138] Ballari S.A., Kuebbing S.E., Nuñez M.A. (2016). Potential Problems of Removing One Invasive Species at a Time: A Meta-Analysis of the Interactions between Invasive Vertebrates and Unexpected Effects of Removal Programs. PeerJ.

[139] Ávila-Thieme M.I., Corcoran D., Pérez Matus A., Wieters E., Navarrete S.A., Marquet P.A., Valdovinos F.S. (2021). Alteration of Coastal Productivity and Artisanal Fisheries Interact to Affect a Marine Food Web. Sci. Rep..

[140] IUCN The IUCN Red List of Threatened Species. https://www.iucnredlist.org/en.

[141] Bonino N., Cossíos D., Menegheti J. (2010). Dispersal of the European Hare, Lepus Europaeus in South America. Folia Zool..

[142] Harcourt-Brown F. (2002). Chapter 1—Biological Characteristics of the Domestic Rabbit (*Oryctolagus cuniculi*). Textbook of Rabbit Medicine.

[143] Myers K., Bults H.G. (1977). Observations on Changes in the Quality of Food Eaten by the Wild Rabbit. Aust. J. Ecol..

[144] Sharp T., Saunders G. (2004). Code of Practice for the Humane Control of Rabbits. Model Code of Practice.

[145] Cooke B.D. (1982). Reduction of Food Intake and Other Physiological Responses to a Restriction of Drinking Water in Captive Wild Rabbits, *Oryctolagus cuniculus* (L.). Wildl. Res..

[146] simonetti J.A. (1989). Microhabitat Use by Small Mammals in Central Chile. Oikos.

[147] Simonetti J.A., Fuentes E.R. (1982). Microhabitat Use by European Rabbits (*Oryctolagus cuniculus*) in Central Chile: Are Adult and Juvenile Patterns the Same?. Oecologia.

[148] Castro S.A., Bozinovic F., Jaksic F.M. (2008). Ecological Efficiency and Legitimacy in Seed Dispersal of an Endemic Shrub (*Lithrea caustica*) by the European Rabbit (*Oryctolagus cuniculus*) in Central Chile. J. Arid Environ..

[149] Alvarado S.A., Figueroa R. R.A., Valladares P., Carrasco-Lagos P., Moreno R. (2015). Aves Rapaces de La Región Metropolitana de Santiago, Chile.

[150] Carevic F.S., Carmona E.R., Muñoz-Pedreros A. (2013). Seasonal Diet of the Burrowing Owl Athene Cunicularia Molina, 1782 (Strigidae) in a Hyperarid Ecosystem of the Atacama Desert in Northern Chile. J. Arid Environ..

[151] Villagrán D. (2016). Selección de Hábitat Por El Pequén (Athene Cunicularia) En La Periferia de La Ciudad de Valdivia, Sur de Chile. Trabajo de Titulación Presentado como Parte de los Requisitos para Optar al Título de Ingeniero en Conservación de Recursos Naturales.

[152] Novoa F., Blanco J. (2020). First Report of a Magellanic Horned Owl (*Buho Magellanicus*) Nesting in a Balcony. Ornitol. Neotropical.

[153] Leveau L.M. (2021). The Harris Hawk (*Parabuteo Unicinctus*) in Urban Areas of Argentina: Arrival in Mar Del Plata City and Green Area Use in Buenos Aires City. Animals.

[154] Figueroa R., González-Acuña D. (2006). Prey of the Harris’s Hawk (*Parabuteo unicinctus*) in a Suburban Area of Southern Chile. J. Raptor Res..

[155] Jansen B.D., Jenks J.A., Jansen B.D., Jenks J.A. (2011). Estimating Body Mass of Pumas (*Puma concolor*). Wildl. Res..

[156] Londoño-Osorio A., Ceballos C.P., Tamayo-Arango L.J. (2020). Anatomical Description of the Origin and Distribution of the Lumbosacral Plexus in One Puma (*Puma concolor*). Anat. Histol. Embryol..

[157] Elbroch L.M. (2017). Pumas: Solitary but Social?. Front. Ecol. Environ..

[158] Karandikar H., Serota M.W., Sherman W.C., Green J.R., Verta G., Kremen C., Middleton A.D. (2022). Dietary Patterns of a Versatile Large Carnivore, the Puma (*Puma concolor*). Ecol. Evol..

[159] Simonetti J.A., Walkowlak A. (1979). Presas de *Tyto Alba* Gray, 1829 (Aves: Strigidae) En El Parque Nacional La Campana. An. Mus. Hist. Nat..

[160] Muñoz-Pedreros A., Gil C., Yáñez J., Rau J.R. (2010). Raptor Habitat Management and Its Implication on the Biological Control of the Hantavirus. Eur. J. Wildl. Res..

[161] Silva S.I. (2005). Posiciones tróficas de pequeños mamíferos en Chile: Una revisión. Rev. Chil. Hist. Nat..

[162] Roach N. (2016). IUCN Red List of Threatened Species: Abrocoma bennettii.

[163] CEA (2006). Abrocoma bennetti (Waterhouse, 1837).

[164] D’Elia G., Pardinas U., Patterson B. (2016). IUCN Red List of Threatened Species: Abrothrix longipilis.

[165] Landaeta-Aqueveque C.A., Robles M.D.R., Cattan P.E. (2007). Helmintofauna Del Roedor *Abrothrix olivaceus* (Sigmodontinae) En Áreas Sub-Urbanas de Santiago de Chile. Parasitol. Latinoam..

[166] Figueroa R.A., Soraya C., Cerda J., Saldivia H. (2000). Roedores, Rapaces y Carnívoros de Aysén.

[167] Lagos San Martín V.O., Rodríguez Cortés J., Cortés Cortés I., Fuenzalida Ávila R., Silva Cabello J., Segovia Niño de Zepeda R., Saavedra Saavedra B. (2009). Catastro y Georeferenciación de Colonias de Chinchillas (Chinchilla laniger) en la Quebrada “Las Gredas” de la Reserva Nacional las Chinchillas: Análisis y Evaluación Espacio-Temporal de la Abundancia Relativa de la Especie en este Sector, Respecto de Otros Registros Históricos y Realizados Recientemente.

[168] Suckow M.A., Stevens K.A., Wilson R.P. (2012). The Laboratory Rabbit, Guinea Pig, Hamster, and Other Rodents.

[169] Iriarte A., Jaksić F.M. (1986). The Fur Trade in Chile: An Overview of Seventy-Five Years of Export Data (1910–1984). Biol. Conserv..

[170] Cunazza P. C., Grimberg P. M., de la Maza M. (2013). CONAF en las Áreas Silvestres Protegidas del Estado: Conservando la Flora y Fauna Amenazada.

[171] MMA *Chinchilla Lanigera* (Molina, 1782). http://especies.mma.gob.cl/CNMWeb/Web/WebCiudadana/ficha_indepen.aspx?EspecieId=762&Version=1.

[172] Edwards M.S. (2009). Nutrition and Behavior of Degus (*Octodon degus*). Vet. Clin. North Am. Exot. Anim. Pract..

[173] MMA (2021). Decreto Supremo N° 44/2021.

[174] Vásquez R., Simonetti J. (1999). Life History Traits and Sensitivity to Landscape Change: The Case of Birds and Mammals of Mediterranean Chile. Rev. Chil. Hist. Nat..

[175] Belmar-Lucero S., Godoy P., Ferrés M., Vial P., Palma R.E. (2009). Range Expansion of *Oligoryzomys longicaudatus* (Rodentia, Sigmodontinae) in Patagonian Chile, and First Record of Hantavirus in the Region. Rev. Chil. Hist. Nat..

[176] Begall S., Burda H., Gallardo M.H. (1999). Reproduction, Postnatal Development, and Growth of Social Coruros, *Spalacopus cyanus* (Rodentia: Octodontidae), from Chile. J. Mammal..

[177] Veitl S., Begall S., Burda H. (2000). Ecological Determinants of Vocalisation Parameters: The Case of the Coruro *Spalacopus cyanus* (Octodontidae), a Fossorial Social Rodent. Bioacoustics.

