# Supplementary material for: European Rabbit Invasion in a Semi-Arid Ecosystem of Chile: How Relevant Is Its Role in Food Webs?
Source: Life (Basel). 2023 Mar 31;13(4):916. doi: 10.3390/life13040916 (PMC10144028; doi:10.3390/life13040916)
Supplement: Supplementary file 1 [file life-13-00916-s001.zip › life-2269990-supplementary.pdf]

## Supplementary material

**Table S1.** Plants in Las Chinchillas National Reserve [62,63,140].

| Scientific name | Common name | IUCN Red List Category |
|---|---|---|
| *Acacia caven* | Roman cassie | Least Concern |
| *Adesmia microphylla* | Pahuen | Least Concern |
| *Adesmia zoolneri* | Ulibarri | Not Evaluated |
| *Adiantum chilensis* | Meyen | Not Evaluated |
| *Alstroemeria dilute* | Peruvian lily | Not Evaluated |
| *Alstroemeria angustifolia* | Inca lily | Not Evaluated |
| *Argylia radiata* | Velvet flower | Not Evaluated |
| *Baccharis linearis* | Chilean little rosemary | Not Evaluated |
| *Bahia ambrosioides* | Chamicilla | Not Evaluated |
| *Bridgesia incisifolia* | Rumpiato | Not Evaluated |
| *Calceolaria* sp. | Capachito | Not Evaluated |
| *Chloraea* sp. | Orchid | Not Evaluated |
| *Cistanthe arenaria* | Renilla | Not Evaluated |
| *Colliguaja odorifera* | Colliguay | Not Evaluated |
| *Cordia decandra* | Carbonillo | Near Threatened |
| *Crukshanskia pumila* | - | Not Evaluated |
| *Cumulopuntia ovata* | Puskaye | Not Evaluated |
| *Dioscorea humifusa* | Huanqui | Not Evaluated |
| *Ephedra andina* | Jointfir | Least Concern |
| *Ephedra rupestris* | Pingo-pingo | Least Concern |
| *Eriosyce curvispinus* | Quisquito | Not Evaluated |
| *Erodium moschatum* | Whitestem filaree | Not Evaluated |
| *Escallonia illinita* | Red escallonia | Not Evaluated |
| *Eulychnia acida* | Copao | Least Concern |
| *Flourensia thurifera* | Ñinquil | Not Evaluated |
| *Gutierrezia resinosa* | Pichanilla | Not Evaluated |
| *Kageneckia oblonga* | Bollén | Least Concern |
| *Krameria cistoidea* | Pacul | Not Evaluated |
| *Leucocoryne coquimbesis* | Huille | Not Evaluated |
| *Leucocoryne purpurea* | Huilli | Not Evaluated |
| *Lithraea caustica* | Litre | Least Concern |
| *Lobelia* sp. | Tupa | Not Evaluated |
| *Lycium chilensis* | Llaullín | Not Evaluated |
| *Maytenus boaria* | Maiten tree | Least Concern |
| *Muehlenbeckia hastulata* | Quilo | Not Evaluated |
| *Mutisia* sp. | - | Not Evaluated |
| *Nassella chilensis* | Chilean needlegrass | Not Evaluated |
| *Oxalis perdicaria* | Wood-Sorrel | Not Evaluated |
| *Pasithea caerulea* | Blue passionflower | Not Evaluated |

| | | |
|---|---|---|
| *Plantago hispidula* | Llantén | Not Evaluated |
| *Porlieria chilensis* | Guayacán | Not Evaluated |
| *Proustia baccharoides* | Olivillo | Not Evaluated |
| *Proustia cinerea* | Huañil | Not Evaluated |
| *Puya berteroniana* | Chagual | Not Evaluated |
| *Quillaja Saponaria* | Quillay | Least Concern |
| *Rodophialla phycelloides* | Red añañuca | Not Evaluated |
| *Schinus latifolius* | False pepper tree | Least Concern |
| *Schinus polygama* | Chilean pepper tree | Least Concern |
| *Schizanthus parvulus* | Butterfly flower | Not Evaluated |
| *Senna cumingii* | Caper | Not Evaluated |
| *Sisyrinchium graminifolium* | Ñuño | Not Evaluated |
| *Sisyrinchium junceum* | Huilmo | Not Evaluated |
| *Stipa plumosa* | Pasto rey | Not Evaluated |
| *Tecoiphilaea violaefora* | - | Not Evaluated |
| *Trevoa trinervis* | Trevo | Not Evaluated |
| *Trichocereus coquimbanus* | Cactus coquimbano | Not Evaluated |
| *Tropaelum azureum* | Perennial blue nasturtium | Not Evaluated |
| *Tropaeolum tricolor* | Chilean nasturtium | Not Evaluated |
| *Vulpia bromoides* | Squirrel Tail Fescue | Not Evaluated |

**Table S2.** Amphibians, arthropods, birds, mammals, and reptiles in Las Chinchillas National Reserve [62,63,140].

| | Scientific name | Common name | IUCN Red List Category |
|---|---|---|---|
| **Amphibians** | *Rhinella arunco* | Concepcion Toad | Near Threatened |
| **Arthropods** | *Acanthinodera cumingii* | Mother of the snake | Not Evaluated |
| | *Grammostola spathulata* | Chilean Rose Tarantula | Not Evaluated |
| **Birds** | *Agriornis lividus* | Great Shrike-tyrant | Least Concern |
| | *Athene cunicularia* | Burrowing Owl | Least Concern |
| | *Bubo magellanicus* | Magellanic Horned Owl | Least Concern |
| | *Colaptes pitius* | Chilean Flicker | Least Concern |
| | *Colorhamphus parvirostris* | Patagonian Tyrant | Least Concern |
| | *Elaenia albiceps* | White-crested Elaenia | Least Concern |
| | *Falco peregrinus* | Peregrine Falcon | Least Concern |
| | *Falco sparverius* | American Kestrel | Least Concern |
| | *Geosita rufpennis* | Minero Cordillerano | Not Evaluated |
| | *Geranoaetus melanoleucus* | Black-chested Buzzard-eagle | Least Concern |
| | *Geranoaetus polyosoma* | Variable Hawk | Least Concern |
| | *Glaucidium nana* | Austral Pygmy-owl | Least Concern |
| | *Metriopelia melanoptera* | Black-winged Ground-dove | Least Concern |
| | *Milvago chimango* | Tiuque | Least Concern |
| | *Mimus thenca* | Chilean Mockingbird | Least Concern |
| | *Muscisaxicola rufivertex* | Rufous-naped Ground-tyrant | Least Concern |
| | *Nothoprocta perdicaria* | Chilean Tinamou | Least Concern |
| | *Ochetorhynchus melanurus* | Crag Earthcreeper | Least Concern |
| | *Parabuteo unicinctus* | Harris's Hawk | Least Concern |
| | *Patagioenas Araucana* | Chilean Pigeon | Least Concern |
| | *Patagona gigas* | Giant Hummingbird | Least Concern |
| | *Pseudasthenes humicola* | Dusky-tailed Canastero | Least Concern |
| | *Pteroptochos megapodius* | Moustached Turca | Least Concern |
| | *Scelorchilus albicollis* | White-throated Tapaculo | Least Concern |
| | *Scytalopus fuscus* | Dusky Tapaculo | Least Concern |
| | *Sephanoides sephaniodes* | Green-backed Firecrown | Least Concern |
| | *Systerulla longirostris* | Band-wined Nightjar | Not Evaluated |

| | | | |
|---|---|---|---|
| | *Theristicus melanopis* | Black-faced Ibis | Least Concern |
| | *Tyto alba* | Common Barn-owl | Least Concern |
| | *Upucerthia dumetaria* | Scale-throated Earthcreeper | Least Concern |
| | *Veniliornis lignarius* | Striped Woodpecker | Least Concern |
| | *Vultur gryphus* | Andean Condor | Vulnerable |
| | *Xolmis pyrope* | Fire-eyed Diucon | Least Concern |
| **Mammals** | *Abrocoma bennetti* | Bennett's Chinchilla Rat | Least Concern |
| | *Abrothrix olivaceus* | Olive Grass Mouse | Least Concern |
| | *Chinchilla lanigera* | Long-tailed Chinchilla | Endangered |
| | *Conepatus chinga* | Molina's Hog-nosed Skunk | Least Concern |
| | *Galictis cuja* | Lesser Grison | Least Concern |
| | *Leopardus colocolo* | Pampas cat | Near Threatened |
| | *Lycalopex culpaeus* | Culpeo fox | Least Concern |
| | *Lycalopex griseus* | Chilla fox | Least Concern |
| | *Octodon degus* | Degu | Least Concern |
| | *Octodon lunatus* | Moon-toothed Degu | Near Threatened |
| | *Phyllotis darwini* | Darwin's Leaf-eared Mouse | Least Concern |
| | *Puma concolor* | Puma | Least Concern |
| | *Spalacopus cyanus* | Cururo | Least Concern |
| | *Thylamys elegans* | Elegant fat-tailed mouse opossum | Least Concern |
| **Reptiles** | *Callopistes maculatus* | Spotted false monitor | Least Concern |
| | *Liolaemus fuscus* | Brown tree iguana | Least Concern |
| | *Liolaemus lemniscatus* | Wreath tree iguana | Least Concern |
| | *Liolaemus nitidus* | Shining tree iguana | Least Concern |
| | *Liolaemus platei* | Braided tree iguana | Least Concern |
| | *Liolaemus zapallarensis* | Zapallaren tree iguana | Least Concern |
| | *Philodryas chamissonis* | Chilean green racer | Least Concern |
| | *Tachymenis chilensis* | Chilean Slender Snake | Near Threatened |

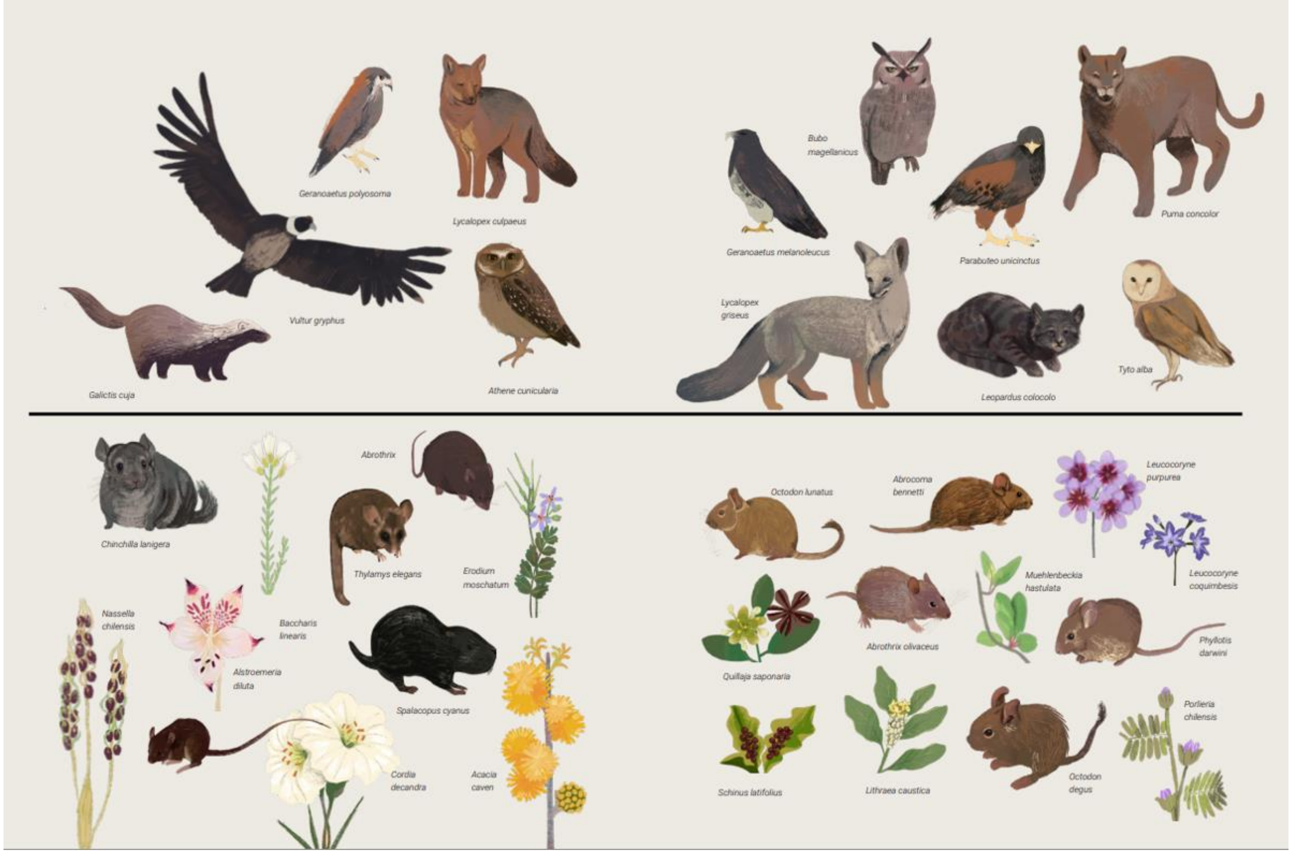


**Figure S1.** Predators, prey, and competitors in Las Chinchillas National food web.

**European rabbit**

- ***Oryctolagus cuniculus***

The European rabbit is a medium-sized mammal that belongs to the family Leporidae, within the order Lagomorpha. Rabbits are characterized by their grayish-brown fur, short tail, powerful hind limbs adapted for running, and large ears [25]. The adult rabbits weigh between 1.5 to 3 kilos, and they could measure 34 to 60 cm from the tail to the head, they do not have a noticeable sexual dimorphism [141], they live as territorial monogamous pairs, or in social groups of up to ten individuals with separate hierarchies for males and females. This species is essentially nocturnal, leaving the burrow in the early evening and returning in the morning, although they can be seen grazing or basking during the day [142]. European rabbits are efficient invaders; they can colonize many habitats because of their high reproductive capacity and adaptability to exploit varied resources [8,143]. Generally, spring is the highest point of their reproductive cycle and autumn the lowest with breeding triggered when pastures green up after rain; furthermore, adult females are receptive throughout the whole year and they could produce 15–40 young a year, but only 1–10% survive past the first year; the surviving juveniles leave their burrows by three weeks of age and reach sexual maturity between four to nine months old [8,26,144,145]. In addition, European rabbits prefer a sandy, hilly, deep, and permeable terrain with shrubs and woody plants to insulate themselves from the heat in arid Regions; these characteristics are necessary for their warrens that can be 3 m deep and 45 m long with tunnels that are about 15 cm in diameter [142,146]. The building of warrens and overgrazing of the vegetation cover when combined with other factors such as

precipitation, strong wind, and steep slopes can increase the risk of soil erosion [8]. Rabbits can consume any vegetable tissue with a minimum of water and nutrients, from grasslands, shrub shoots, tree barks, fruiting trees, to cacti, tubers, rhizomes, and flowers [147,148]. In the north-central zone of Chile, rabbits feed mainly on the foliage and seeds of *Lithraea caustica* [149], *Quillaja saponaria* and *Schinus latifolius* foliage, *Porlieria chilensis* shoots, *Leucocoryne purpurea* and *L. coquimbesis* flowers, *Muehlenbeckia hastulata* leaves and exotic plants (e.g., *Vulpia bromoides*) [16]. In LChNR part of its diet are the plants *Oxalis perdicaria*, *Plantago hispidula, Schizanthus parvulus, Senna cumminggi, Tropaeolum azureum*; *Flourensia thurifera,* and *Maytenus boaria* (M. Ortiz CONAF, pers. comm.).

***Native predators***
Within Las Chinchillas National Reserve, twelve species are predators that consume rabbits; they correspond to native birds of prey, such as hawks, eagles, and owls, and native carnivores such as felines, canids, and a mustelid, which are described below.

- ***Athene cunicularia***

The Burrowing Owl is a raptor that belongs to the family Strigidae, within the order Strigiformes. It shows a wide distribution in the Americas, from Canada to the south of Chile and Argentina [150,151]. In Chile it can be found from the Arica y Parinacota Region to the Magallanes Region [150]. Burrowing Owl's total length goes from 18 to 30 cm, it weighs between 150 to 370 grams, and has a wingspan of 50 to 68 cm [68,152]. This species is characterized by consuming preeminently invertebrates all year [68]. Between 75 to 95% of the total prey are *Grammostola spathulata* [67] and insects from the orders Coleoptera, Dermaptera, Hemiptera, Hymenoptera, Lepidoptera, Odonata, Diptera, and Orthoptera [49]. Rodents and anurans are important as well, but their consumption level fluctuate seasonally, suggesting that the Burrowing Owl is a seasonally opportunistic species with a diurnal and crepuscular activity [49,67,68,152], including birds of the order Passeriformes [27,49,66,67]. The lower consumption of vertebrates over invertebrates is due to the vertebrates' more significant contribution to their biomass [68]. In Aucó, Torres et al. (1994) found that the Burrowing Owl preyed indistinctly on juveniles and adults of the rodent *Phyllotis darwini* [66]. Also, it consumes other native species such as the rodents *Abrocoma bennetti, Abrothrix longipilis, Abrothrix olivaceus, Chinchilla lanigera, Octodon degus, Oligoryzomys longicaudatus,* and *Spalacopus cyanus,* the marsupial *Thylamys elegans*, the lizard *Liolaemus fuscus*, and the exotic lagomorph *Oryctolagus cuniculus*, also consumes insects and arachnids [49,66,67].

**Table S3.** Records of prey in *Athene cunicularia* diet between 1973 and 2004, in percentage of biomass of their prey, based on pellet analyses made in different studies throughout time.

| Prey | **Schlatter et al. 1980** [49] | **Jaksic et al. 1981** [27] | **Torres-Contreras et al. 1994** [66] | **Cruz-Jofré & Vilina 2014** [67] |
|---|---|---|---|---|
| Arthropods | 79.7% | 79.2% | 89.8% | 89.2% |
| Rodents | 16% | 16.1% | 5.9% | 1% |
| *Oryctolagus cuniculus* | 0.1% | 0.1% | 0.1% | 0.3% |
| Marsupials | 1.8% | 0.4% | 7% | 2.2% |
| Anurans | 2.9% | 3% | 3.8% | - |

| | | | | |
|---|---|---|---|---|
| Birds | 0.9% | 1% | 0.3% | 5.6% |
| Reptiles | 0.03% | 0.2% | 0.1% | 1.1% |
| **Study's date** | August 1973 – April 1974 | 1973 - 1979 | 1987 – 1993 | 2004 |
| **Study site** | La Dehesa, Metropolitan Region (MR) | La Dehesa and Los Dominicos, MR | Las Chinchillas National Reserve, Coquimbo Region | Pingüino de Humboldt National Reserve, Atacama Region |

- ***Bubo magellanicus***

The Magellan Great Horned Owl or Magellanic Owl is a nocturnal bird of prey that belongs to the family Strigidae, within the order Strigiformes. It has a wide distribution in the southern portion of South America, from the center of Peru and west of Bolivia to the south of Chile and Argentina [150,153]. The Magellanic Owl is the biggest Chilean owl and can weigh from 517 to 1500 grams [150,153]. Thirty percent of their prey are nocturnal as well, with high consumption of mammals, such as rodents and lagomorphs [68]. In central Chile, their most frequent prey are the small mammals *Abrocoma bennetti*, *Abrothrix longipilis*, *Abrothrix olivaceus*, *Octodon degus*, *Octodon lunatus*, *Oligoryzomys longicaudatus*, *Oryctolagus cuniculus*, *Phyllotis darwini*, *Spalacopus cyanus*, and *Thylamys elegans;* birds such as, *Nothoprocta perdicaria* and other of the order Passeriformes, and a variety of insects and arachnids [69,70,84].

**Table S4.** Records of prey in the *Bubo magellanicus* diet between 1973 and 2001, in percentage of biomass of their prey, based on pellet analyses made in different studies throughout time.

| **Prey** | **Jaksic et al. 1981** [27] | **Yáñez et al. 1978** [84] | **Muñoz-Pedreros et al. 2017** [70] |
|---|---|---|---|
| Arthropods | - | 77.5% | 0.2% |
| Rodents | 69.3% | 12.6% | 52% |
| *Oryctolagus cuniculus* | 15.8% | 1.5% | 42.3% |
| Marsupials | 3.5% | - | 0.3% |
| Birds | 11.4% | 8.4% | 5% |
| **Study's date** | 1973 - 1979 | September 1976 | September 2000 – December 2001 |
| **Study site** | La Dehesa and Los Dominicos, MR | Tinguiririca river, O´Higgins Region | Lago Peñuelas National Reserve, Valparaíso Region |

- ***Galictis cuja***

The Lesser grison is a mammal of the family Mustelidae within the order Carnivora. It is widely distributed throughout the Americas, from the southern half of Mexico, passing southward through Peru, Bolivia, Brazil, Paraguay, Uruguay, and extending to the Chilean and Argentine Patagonia [72]. It measures between 44 to 76 cm in length and the adult weight is around 1.88 kg, with a slight sexual dimorphism in favor of males. Regarding their behavior, they have solitary and aggressive habits and are active during the day. Moreover, they are very agile, can execute quick zig-zag movements, and build long galleries [71]. In north-central Chile, the Lesser grison usually inhabits evergreen shrublands, consumes rodents, birds of the order Passeriformes, lizards, and snakes [71]. Ebensperger et al. (1991), from feces collected in 1988-1989, found that the Lesser grison diet was based on the mammals *Abrocoma bennetti* (2.9%), *Abrothrix longipilis* (8.8%), *Oligoryzomys longicaudatus* (2.9%), *Oryctolagus cuniculus* (26.5%), and *Phyllotis darwini* (5.9%); the snake *Philodryas chamissonis* (14.7%), and several birds (2.9%) [85].

- ***Geranoaetus melanoleucus***

The Black-chested Eagle or Grey Eagle-buzzard is a diurnal raptor that belongs to the family Accipitridae, within the order Falconiformes. It has a wide distribution in South America, from Colombia to Tierra del Fuego, living in open spaces from sea level to 2200 meters altitude or even higher [150, 76]. This species is the biggest Chilean falconiform, with a wingspan between 150 to 183 cm and a total length between 65 to 80 cm; moreover, female adults could weigh 3170 grams, whereas adult males can only reach up to 2610 grams [150,75]. The activity periods of these eagles are bimodal, peaking in the midmorning and mid-evening, and flying takes up most of this time, except in winter [74]. The Black-chested Eagle principally consumes mammals, being almost 70% of their prey, mostly *Octodon degus, Abrocoma bennetti*, and *Oryctolagus cuniculu*s. It also consumes *Abrothrix longipilis, Abrothrix olivaceus, Oligoryzomys longicaudatus, Phyllotis darwini*, and *Thylamys elegans* [73,74,76]. Birds represent about 12% of their total prey, including *Nothoprocta perdicaria*, *Metriopelia melanoptera*, *Colaptes pitius*, *Schelorchilus albicollis*, and other birds of the order Passeriformes [74]. Reptiles represent about the remaining 18% of their diet, and most of them correspond to the snake *Philodryas chamissonis*. Insects are group less consumed by Black-chested Eagle [74].

**Table S5.** Records of prey in the *Geranoaetus melanoleucus* diet between 1973 and 1989, in percentage of biomass of their prey, based on pellet analyses made in different studies throughout time.

| Prey | **Jaksic et al. 1981** [27] | **Jiménez & Jaksic 1989** [74] | **Pavez & Jiménez 1992** [54] |
|---|---|---|---|
| Rodents | 75.9% | 52% | 14.1% |
| Reptiles | 4.1% | 12% | 3.4% |
| *Oryctolagus cuniculus* | 18.8% | 18% | 81.7% |
| Birds | 1.2% | 16% | 0.8% |
| Marsupials | - | 2% | - |
| **Study's date** | 1973 - 1979 | August 1984 - August 1985 | December 1987 – March 1989 |
| **Study site** | La Dehesa and Los Dominicos, MR | San Carlos de Apoquindo, MR | San Carlos de Apoquindo, MR |

- ***Geranoaetus polyosoma***

The Variable Hawk is a bird of prey with diurnal activity that belongs to the family Accipitridae of the order Falconiformes [77,78]. This raptor has a wide distribution within South America and is located from the north of the central mountain range of Colombia to Cabo de Hornos, and from the seacoasts of Chile to Argentina, reaching the Malvinas and Alejandro Selkirk island [77]. The Variable Hawk has medium size with a wingspan of 110 cm; as to sexual dimorphism, the male adults weigh 690 to 1134 grams and measure 450 to 530 mm, whereas female adults weigh 876 to 1417 grams and could measure up to 630 mm [102]. The Variable Hawk feeds on small vertebrates and invertebrates, particularly arthropods, reptiles (*Liolaemus platei*), and small mammals such as *Abrocoma bennetti, Abrothrix olivaceus, Oligoryzomys longicaudatus,* and *Phyllotis darwini* [77,150]. A study made between 1973 and 1979 in La Dehesa and Los Dominicos, MR, based on the analysis of their collected pellets, found that rodents represented 89.3%, reptiles 0.3%, birds 3.3%, marsupials 1% of the diet, whereas *O. cuniculus* corresponded to 6.1% of their diet [28].

***Leopardus colocolo***

The Pampas cat belongs to the family Felidae of the order Carnivora. Is distributed from Ecuador to Matto Grosso in Brazil by the north to the Chilean and Argentinian Patagonia by the south [81]. This species can be found in open terrains and grassland areas and its size is similar to that of the domestic cat but is more robust. It measures between 52 to 70 cm with a short tail (22-33 cm), it weighs 2 to 8 kg, and the females are smaller than the males [71]. This feline is strict carnivore, their prey are mainly small mammals such as rodents (e.g., *Phyllotis darwini*), marsupials, birds, reptiles, and more recently rabbits [71,80].

***Lycalopex culpaeus***

The Culpeo fox belongs to the family Canidae of the order Carnivora. It is found throughout the Andes ranges from Colombia to Tierra del Fuego [83]. In Chile, the Culpeo fox is the biggest canid, with a body length of 50 to 100 cm, and weighs 5 to 13 kilos [47]. It is a solitary animal with nocturnal-crepuscular activity [71]. This omnivorous fox feeds from invertebrates to small and medium vertebrates, including coleopterans, orthopterans, arachnids (e.g., *Grammostola spathulata*), and mammals such as *Abrocoma bennetti, Abrothrix longipilis, Abrothrix olivaceus, Octodon degus, Oligoryzomys longicaudatus, Oryctolagus cuniculus, Phyllotis darwini,* and *Thylamys elegans;* snakes (e.g., *Philodryas chamissonis*) carrion and plants (e.g., *Porlieria chilensis*) [83,85]. Rubio et al. (2013) reported that the Culpeo fox is now a major consumer of European rabbit in central Chile [56].

**Table S6.** Records of prey in *Lycalopex culpaeus* diet between 1973 and 2015, in percentage of biomass of their prey, based on feces analyses made in different studies throughout time.

| **Prey** | **Jaksic et al. 1980** [47] | **Yáñez & Jaksic 1978** [84] | **Ebensperger et al. 1991** [85] | **Zúñiga & Fuenzalida 2016** [83] | **Osorio et al. 2020** [82] |
|---|---|---|---|---|---|
| Reptiles | 4.7% | - | 8.7% | 8% | - |
| Birds | 5.6% | 9.5% | 14.5% | 12% | 8% |
| Rodents | 70% | 85.7% | 53.7% | 64% | 75% |
| *Oryctolagus cuniculus* | 19.7% | 4.8% | 21.7% | 8% | 20% |
| Marsupials | - | - | 1.4% | - | - |
| **Study's date** | 1973 – 1978 | 1976 – 1978 | 1988 – 1989 | October 2014 – February 2015 | 2015 |
| **Study site** | La Dehesa, La Disputada, Los Dominicos & Lagunillas, MR | Fundo Santa Laura, MR | El Pangue, MR | Arica y Parinacota Region, La Araucanía Region & Los Ríos Region | Río Cipreses NR, O´Higgins Region |

***Lycalopex griseus***

The Chilla fox is another member of the family Canidae of the order Carnivora. It can be found in the southern of Peru, Argentina, and Chile [87]. In Chile, it is naturally distributed throughout the country, but in Tierra del Fuego is an introduced species [11]. It is a medium-sized fox, which measures between 40 to 60 cm, plus 30 to 36 cm for the tail; its weight can reach 4 kg for males and 3.5 kg for females [71]. It is omnivorous with a very broad diet, feeding on arthropods (e.g., *Acanthinodera cumingii),* reptiles (e.g., *Liolaemus nitidus),* vegetal material, carrion, and small mammals such as *Abrocoma bennetti, Abrothrix*

*longipilis, Abrothrix olivaceus, Octodon degus, Octodon lunatus, Oligoryzomys longicaudatus, Oryctolagus cuniculus, Phyllotis darwini,* and *Thylamys elegans* [86,87].

**Table S7.** Records of prey in *Lycalopex griseus* diet between 1973 and 2001, in percentage of biomass of their prey, based on feces analyses made in different studies throughout time.

| **Prey** | **Jaksic et al. 1980** [47] | **Yáñez & Jaksic 1978** [84] | **Muñoz-Pedreros et al. 2018** [87] |
|---|---|---|---|
| Rodents | 93.4% | 100% | 44.7% |
| *Oryctolagus cuniculus* | 3.3% | - | 52.2% |
| Birds | 3.3% | - | 2.3% |
| Reptiles | - | - | 0.2% |
| Insects | - | - | 0.2% |
| **Study's date** | 1973 – 1978 | 1976 – 1978 | 2001 |
| **Study site** | La Dehesa, La Disputada, Los Dominicos, Lagunillas & Fundo Santa Laura, MR | Fundo Santa Laura, MR | Lago Peñuelas NR, Valparaíso Region |

- ***Parabuteo unicinctus***

The Harris Hawk is a diurnal raptor that belongs to the family Accipitridae within the order Falconiformes. It inhabits the semi-open habitat of southern North America, Central America, and South America. In Chile is widely distributed, mostly in Arica y Parinacota Region, and from Atacama Region to Aysen Region [150,155]. This raptor presents sexual dimorphism, the adult male weighing 550-870 g and the adult female 820-1300 g, with a wingspan of 100 to 125 cm [156]. The Harris Hawk preys mostly on small mammals [155]. Throughout the years, there has been a change in their diet from 1980 to 2006, where the percentage of *Octodon degus* has been diminishing, while the percentage of *Oryctolagus cuniculus* has been increasing [55]. Common prey recorded for the Harris Hawk in Las Chinchillas National Reserve are the small mammals *Abrocoma bennetti, Abrothrix longipilis, Abrothrix olivaceus, Oligoryzomys longicaudatus, Phyllotis darwini, Spalacopus cyanus, and Thylamys elegans;* the bird *Pteroptochos megapodius;* and the snakes *Philodryas chamissoni,* and *Tachymenis chilensis* [28,48,156].

**Table S8.** Records of prey in the *Parabuteo unicinctus* diet, in percentage of biomass of their prey, based on pellet analyses made in different studies throughout time.

| **Prey** | **Jaksic et al. 1980** [48] | **Pávez et al. 2010** [55] |
|---|---|---|
| Rodents | 91.9% | 16% |
| Reptiles | 5.2% | - |
| *Oryctolagus cuniculus* | 1.2% | 84% |
| Birds | 1.7% | - |
| **Study site** | La Dehesa, Los Dominicos, MR | La Dehesa, MR |

***Puma concolor***

The Puma belongs to the family Felidae of the order Carnivora. It is distributed all over the American continent, and is the biggest feline in Chile, with a body length between 105 to 180 cm plus 60 to 90 cm for the tail [71]. It has sexual dimorphism, with adult males (53 to

100 kg) being bigger than the adult females (34 to 70 kg) [88]. The Puma is a solitary and territorial, but a social animal with nocturnal-crepuscular activity because sometimes aggregates at kills made by conspecifics for short periods [71,157-159]. Pumas are successful generalist predators, and due to their big size and a wide range of action, they have a high energy demand [160]. Their diet is very diverse depending on the environments that they inhabit; thus, in Las Chinchillas National Reserve they consume *Abrothrix longipilis, Abrothrix olivaceus, Lycalopex culpaeus, Oligoryzomys longicaudatus,* and *Oryctolagus cuniculus* [82,88].

- ***Tyto alba***

  The Barn Owl is a nocturnal raptor that belongs to the family Tytonidae within the order Strigiformes [150] that reaches a length between 38 to 43 cm with a wingspan between 68 to 108 cm [89]. This species is distributed from southern North America to Chile, and it is considered beneficial for agricultural and livestocking activity because it contributes to the equilibrium in natural ecosystems owing to their specialized diet on small mammals, preferably nocturnal [68,89,161], although it also consumes birds of the order Passeriformes [89]. Its most common prey is *Phyllotis darwini*, but it also consumes *Abrocoma bennetti, Abrothrix olivaceus, Octodon degus, Octodon lunatus, Oligoryzomys longicaudatus, Oryctolagus cuniculus*, *Spalacopus cyanus,* and *Thylamys elegans;* birds and coleopterans [46,86,91,161,162].

**Table S9.** Records of prey in the *Tyto alba* diet between 1974 and 2001, in percentage of biomass of their prey, based on pellet analyses made in different studies throughout time.

| **Prey** | **Jaksic & Yáñez 1979** [46] | **Ebensperger et al. 1991** [85] | **González et al. 2004** [89] |
|---|---|---|---|
| Rodents | 91.3% | 79.8% | 85.1% |
| Marsupials | 3.4% | 16.4% | - |
| Birds | 4.2% | 0.8% | - |
| *Oryctolagus cuniculus* | - | 3% | 12.5% |
| Insects | - | - | 2.4% |
| **Study's date** | 1974 - 1978 | 1988 - 1989 | November 1999 - December 2001 |
| **Study site** | San Felipe & Lago Peñuelas NR, Valparaíso Region; Los Dominicos, Las Vizcachas & Talagante, MR; San Fernando, O´Higgins Region | El Pangue, MR | Campus Chillán, Universidad de Concepción, Biobío Region |

- ***Vultur gryphus***

  The Andean Condor belongs to the family Cathartidae, within the order Falconiformes [150]. This scavenging raptor measures between 105 to 130 cm long, with a wingspan between 300 to 320 cm; male adults can weigh 11 to 15 kilos, whereas female adults 8 to 11 kilos [92,150]. This makes this species the biggest scavenger bird in the world, which inhabits the Andes Mountains from 5°N to 55°S latitude [93]. Like other scavengers, the Andean Condor has been able to adapt to changing environments, modifying its dietary behavior, which depends on resource availability in the different types of ecosystems present (natural or anthropized) [93]. Duclos et al. (2020) recorded the diet of the Andean Condor in central Chile, which allows inferring their diet in the Las Chinchillas National Reserve as consisting of carrion of *Galictis cuja*, *Lycalopex culpaeus*, *Oryctolagus cuniculus*, and *Puma concolor* [93].

**Native small mammals**

In Las Chinchillas National Reserve, *Oryctolagus cuniculus* coexists with several rodent species and one marsupial species, which have different behaviors and food habits; these are described below.

***Abrocoma bennetti***

Bennet´s Chinchilla rat belongs to the family Abrocomidae, within the order Rodentia [67] and measures 325 to 405 mm total length with 195 to 275 grams [94]. This rodent has mostly nocturnal activity, and lives in high-cover shrublands [164]. The Chinchilla rat can be found from Atacama to Maule Region; this wide distribution allows it to be considered of Least Concern by IUCN [164]. It is an herbivorous animal that feeds on conductive tissue of trees and shrubs, such as *Acacia caven* and *Lithraea caustica*; it also eats herbs and subshrubs such as *Bridgesia incisaefolia, Cordia decandra, Discorea humifusa, Ephedra andina, Flourensia thurifera,* and *Nassella chilensis* [94,165].

***Abrothrix longipilis***

The Long-haired grass mouse is a rodent that belongs to the family Cricetidae. It measures 13 to 15 cm long, plus 8 to 9 cm for the tail and its weight fluctuates between 30 to 50 grams [94,163]. In Chile, it lives from the Coquimbo Region to Tierra del Fuego Island in Magallanes Region, thus inhabiting a variety of ecosystems. It has nocturnal activity [94]. This species is listed as of Least Concern by IUCN, despite its population apparently decreasing [166]. The Long-haired grass mouse is omnivorous (both frugivorous and insectivorous) but is more frugivorous during the summer than in other seasons [99]. Part of its diet are the plants *Alstromeria angustifolia, Alstroemeria diluta,,* and *Proustia baccharoides;* and a variety of arthropods [95].

- ***Abrothrix olivaceus***

The Olive Grass Mouse also belongs to the family Cricetidae. It is widely distributed in Chile, from the far north of the country to the Aysén Region. It inhabits a variety of ecosystems, such as grasslands, shrublands, forests, stony areas, and montane sites [167]. It is small, with a total body length, including the tail, of 17 cm and a weight of 30.4 grams [96]. It presents continuous activity, which means it is both diurnal and nocturnal [168]. The Olive Grass Mouse is omnivorous [163] and consumes herbaceous plants and fungi; invertebrates such as insects, spiders, and earthworms; and seeds such as those of *Acacia caven*, together with fruits, grains, and needles [94,96].

***Chinchilla lanigera***

The Long-tailed chinchilla (also called Coastal chinchilla) is a rodent that belongs to the family Chinchillidae, endemic to Chile [169]. Currently, its distribution is restricted to a few, small, and fragmented colonies, it is distributed along the Cordillera de La Costa and the Cordillera de Los Andes foothills in the Coquimbo Region, specifically in the Las Chinchillas National Reserve (Illapel) and its surroundings. Las Chinchillas National Reserve is the only area that protects colonies of this species together with its ecosystem [170]. There is an isolated population 100 km north of Coquimbo. Also, it has been recorded in the Atacama and Antofagasta Regions. In the latter, between the Coloso sector, south of Antofagasta, to Tal-Tal. From sea level to 3,000 m asl [98,169]. The total population of chinchillas in wild conditions may range from 2440 to 11705 individuals and their colonies range between 0.9

and 10.7 individuals/ha. Lower values are currently estimated, because it is roughly calculated that only 6% of the total area of said Reserve is being occupied by the chinchilla (this is given by the percentage of sectors with suitable habitat, rather than by the population dynamics of this species) [98,169]. More than 20 million specimens that were killed in Chile alone at the beginning of the 20th century [171], thus populations has been reduced to a fraction of the original distribution and is under continuous pressure due to illegal hunting and quality reduction of its habitat [98]. The Long-tailed chinchilla has been classified as Endangered by CONAF [172], as Endangered for northern and central Chile under the Hunting Law and Regulations [173], and as Endangered by IUCN since 2016, this latter due to an ongoing population decline estimated at more than 90% over the last three generations (15 years) [98]. The Long-tailed chinchilla is considered colonial and nocturnal, which preferentially inhabits mountain habitats, and measures from 22.5 to 26 cm from head to trunk and wit a tail from 17 to 18 cm long [94,169]. In addition, the Long-tailed chinchilla is smaller in Las Chinchillas National Reserve and presents less sexually dimorphic features than domestic chinchillas: males weigh 412 ± 9 grams (369-493 g) and females 422 ± 7 g (range, 379-450 g) [97]. The Long-tailed chinchilla coexists with a diverse set of rodents such as *Abrocoma bennetti*, *Abrothrix longipilis*, *Abrothrix olivaceus*, and *Octodon degus* [94]. The main predators of chinchillas in the Coquimbo Region are the foxes *Lycalopex culpaeus* and *Lycalopex griseus* [94]. The main plant species consumed by the Long-tailed chinchilla are the perennial graminoid *Nassella chilensis*, and secondarily *Adiantum chilense, Bridgesia incisifolia, Heliotropium stenophyllum,* and *Lobelia polyphylla* [98].

***Octodon degus***

The Degu is a rodent belonging to the family Octodontidae [99]. It is only found in Chile, between the Atacama and Maule Regions. It measures about 30 cm, of which a third corresponds to the tail, and weighs about 189 grams. It is diurnal and colonial in habit [94]. The degu is herbivorous and in desertic habitats does not ingest water directly but gets it from its food [163,174]. It feeds on the foliage and seeds of *Baccharis linearis* [99], *Schinus latifolius* [16], *Acacia caven*, *Erodium cicutarium, Porlieria chilensis* [100], *Lithraea caustica*, *Muehlenbeckia hastulata*, *Trevoa trinervis* [101], *Colliguaja odorífera, Kageneckia oblonga, Quillaja saponaria*, and *Vulpia bromoides* [102].

***Octodon lunatus***

The Moon-toothed degu is an endemic Octodontid rodent of Chile that is present in the Regions of Coquimbo, Valparaíso, and Metropolitan. It measures 18.8 cm plus 17.2 cm for its tail [94] and its weight is around 130 to 253 g [103]. It is presumed that its period of activity is crepuscular or nocturnal [94]. Its status is Vulnerable according to the conservation category of Chile [175], and Vásquez and Simonetti (1999) consider that it has a high index of sensitivity to changes in its habitat, such as soil perturbation [179]. It is mainly herbivorous, consuming leaves, seeds, and grasses, and a very small proportion of insects [94,163].

***Oligoryzomys longicaudatus***

The Long-tailed rice mouse is a Cricetid rodent found in northeastern Argentina and in nearly all of Chile, from sea level to 2,000 meters [94]. This species is characterized for having an extremely long tail, 14 cm, almost twice the length of its body (7.6 cm) and weighs from 22 to 35 grams [96,177]. It is nocturnal and solitary. It is herbivorous, mainly granivorous

[89,163], and the most consumed seeds are those of *Acacia caven, Chloraea* sp., and *Erodium moschatum*. It also consumes mosses and arthropods [94,95].

***Phyllotis darwini***

Darwin's leaf-eared mouse is a Cricetid rodent endemic of Chile, it lives from the town of Paposo in the Antofagasta Region, to the province of Malleco in the Araucanía Region. From sea level to 2,000 masl, in semiarid thorn scrub, and in scattered desert habitats [94,96]. It has an adult weight of about 50 grams and a total length of 21.5 cm [96]. It is nocturnal and herbivorous, especially during the spring, while in summer it becomes more granivorous [163]. It consumes plant tissue, seeds, and insects, but also leans towards consuming the foliage of *Baccharis linearis* and the seeds of *Erodium moschatum* and of *Porlieria chilensis* [94,99].

***Spalacopus cyanus***

The coruro is a medium-sized fossorial rodent belonging to the family Octodontidae (Iriarte 2008) and is endemic to central Chile, from Caldera in the Atacama Region to the Ñuble Region. From sea level to 3,400 m asl [178,179]. It measures 14.4 cm from head to trunk and its tail measures 4.4 cm, weighing 70 to 120 g. It is fossorial, gregarious and lives in galleries which it excavates in the ground [94]. It is herbivorous [163], feeding on roots and bulbs of Iridaceae such as *Sisyrinchium graminifolium*, lilac species like *Leuchocoryne purpurea*, and subterranean bulbs including those of *Alstromeria angustifolia*, *Alstroemeria diluta*, *Dioscorea humifusa*, and *Leuchocoryne coquimbesis* [94].

***Thylamys elegans***

The Elegant mouse opossum is a marsupial that belongs to the family Didelphidae [163], which is medium-sized of the order Didelphimorphia, as it measures between 186 to 277 cm in total length and weighs between 26 and 35 grams [94]. It inhabits the north-central zone of Chile. Exists a relict population at the mouth of the Loa River in the Antofagasta Region and from the Atacama Region to the Biobío Region, from sea level to 3,500 masl [94]. The Elegant mouse opossum is nocturnal and a climber, having a prehensile tail and an opposable thumb for it [94]. It is mainly an insectivorous species [163], feeding especially on Lepidoptera and barely on Coleoptera, Diptera, and Orthoptera, as well as on spiders, although it has also been observed consuming bird eggs, fruits of *Erodium moschatum* and *Lycium chilense*, and grass seeds [94,99].
